# *Pv*GAMA reticulocyte binding activity: predicting conserved functional regions by natural selection analysis

**DOI:** 10.1186/s13071-017-2183-8

**Published:** 2017-05-19

**Authors:** Luis A. Baquero, Darwin A. Moreno-Pérez, Diego Garzón-Ospina, Johanna Forero-Rodríguez, Heidy D. Ortiz-Suárez, Manuel A. Patarroyo

**Affiliations:** 1 Molecular Biology and Immunology Department, Fundación Instituto de Inmunología de Colombia (FIDIC), Carrera 50 No. 26-20, Bogotá DC, Colombia; 2 PhD Programme in Biomedical and Biological Sciences, Universidad del Rosario, Carrera 24 No. 63C-69, Bogotá DC, Colombia; 3 Basic Sciences Department, School of Medicine and Health Sciences, Universidad del Rosario, Carrera 24 No. 63C-69, Bogotá DC, Colombia

**Keywords:** Adhesin protein, *Plasmodium vivax*, Genetic diversity, Conserved functional region, Reticulocyte binding activity

## Abstract

**Background:**

Adhesin proteins are used by *Plasmodium* parasites to bind and invade target cells. Hence, characterising molecules that participate in reticulocyte interaction is key to understanding the molecular basis of *Plasmodium vivax* invasion. This study focused on predicting functionally restricted regions of the *P. vivax* GPI-anchored micronemal antigen (*Pv*GAMA) and characterising their reticulocyte binding activity.

**Results:**

The *pvgama* gene was initially found in *P. vivax* VCG-I strain schizonts. According to the genetic diversity analysis, *Pv*GAMA displayed a size polymorphism very common for antigenic *P. vivax* proteins. Two regions along the antigen sequence were highly conserved among species, having a negative natural selection signal. Interestingly, these regions revealed a functional role regarding preferential target cell adhesion.

**Conclusions:**

To our knowledge, this study describes *Pv*GAMA reticulocyte binding properties for the first time. Conserved functional regions were predicted according to natural selection analysis and their binding ability was confirmed. These findings support the notion that *Pv*GAMA may have an important role in *P. vivax* merozoite adhesion to its target cells.

**Electronic supplementary material:**

The online version of this article (doi:10.1186/s13071-017-2183-8) contains supplementary material, which is available to authorized users.

## Background

*Plasmodium vivax* is a human malaria-causing parasite whose eradication is a priority on the international health agenda [1]. As a strategy for eradicating this species, several research groups have focused their efforts on developing a vaccine, as vaccination has been successful at controlling and eradicating other infectious diseases [2].

It has been suggested that vaccines should consist of key proteins or their fragments used by infectious agents to bind to the target cells [3, 4]. Hence, knowledge of proteins expressed by the parasite at the end of its intra-erythrocyte life-cycle, especially those interacting with red blood cells (RBC), should prove most suitable as candidate vaccine components.

Current efforts to develop an anti-malarial vaccine have mainly focused on *P. falciparum*, given the availability of robust in vitro culturing techniques for this parasite (currently unavailable for *P. vivax*) which has led to a large-scale identification of genes [5], transcripts [6] and proteins [7]. This information has led to an improved understanding of the molecules involved in *P. falciparum* merozoite invasion of erythrocytes. For example, several adhesin molecules have been described in the apical organelles (rhoptries and micronemes), that facilitate interaction with cell receptors and promote parasite internalisation within the target cell [8]. Several of these proteins are immunogenic and are being evaluated as vaccine candidates in clinical studies [9]. The GPI-anchored micronemal antigen **(**GAMA) represents one apical protein that has an adhesive role in *Plasmodium* and *Toxoplasma. Plasmodium falciparum* GAMA (*Pf*GAMA) binds to human erythrocytes, an interaction mediated by its binding region which is located in the amino terminal sequence, and is involved in the sialic acid-independent invasion pathway [10]. On the other hand, GAMA knockouts of *T. gondii* (*Tg*GAMA) show a reduction in the ability of tachyzoites to attach to the host cell during invasion as well as a delay in the time to death in an in vivo model, suggesting a function during parasite adhesion and invasion [11].

Unfortunately, basic *P. vivax* research has been delayed mainly due to the parasite’s preference for invading reticulocytes which are difficult to obtain in the high percentages needed for propagating *P. vivax* in vitro [12, 13]. However, it has been possible to characterise several molecules forming part of the parasite’s selective human reticulocyte invasion route, such as reticulocyte binding proteins (RBPs) [14, 15], merozoite surface protein 1 (MSP-1) [16], some proteins from the tryptophan-rich antigen (TRAg) family [17] and the recently described rhoptry neck protein 5 (RON5) [18]. Some of these contain specific binding regions that have been identified using several strategies, such as mapping using peptides labelled with radioactive iodine, ELISA, flow cytometry or rosetting assays. However, these methodologies are laborious when large molecules must be analysed. Furthermore, sometimes it is not known whether these regions are polymorphic between isolates, which would be counterproductive for the development of a broadly protective vaccine.

A new strategy has recently been proposed for identifying selection signals and that enables the determination of conserved antigens or those having potential functional regions [19]. Cornejo et al. [20] and Garzón-Ospina et al. [19] identified natural selection signals in *P. vivax* genes when analysing the sequences of five genomes from different locations [21]. These results were supported by earlier studies, increasing the number of sequences analysed [22–24]. This type of analysis could therefore provide a viable approach for selecting conserved antigens that are subject to functional restrictions. However, no experimental evidence has been produced to support such approach.

Given the importance of conserved functional region prediction and the role of adhesin proteins during host-parasite interaction, and considering the interesting features displayed by GAMA in other apicomplexa, the present study aimed at characterising *P. vivax* VCG-I strain GAMA functional regions by selection signal prediction and then determine the role of such regions in binding to reticulocytes.

## Methods

### An approach to GAMA genetic diversity and evolutionary forces

Evolutionary methods compare the non-synonymous mutations rate (d_N_, mutations altering protein sequences) to the synonymous mutations rate (d_S_, those encoding the same amino acid) in the search for natural selection signals. Deleterious mutations are usually removed from populations by negative natural selection (d_N_ < d_S_ or ω < 1). Regions displaying this kind of selection might have functional/structural importance, maintaining high sequence conservation between species [25]. On the other hand, mutations having an adaptive advantage (or a beneficial role) are fixed in a population by positive natural selection (dN > dS or ω > 1). Taking the above into account, functional regions could be predicted by evolutionary approaches [19]. *pvgama* gene DNA sequences from 6 *P. vivax* strains (VCG-I, Sal-I, Brazil-I, India-VII, Mauritania-I and North Korea [21]) and 5 phylogenetically-related species (*P. cynomolgi*, *P. inui*, *P. fragile*, *P. knowlesi* and *P. coatneyi*) [26] were obtained by tblastn (except for VCG-I) from the whole-genome shotgun contigs (wgs) NCBI database for assessing genetic diversity and evolutionary forces regarding GAMA. The MUSCLE algorithm [27] was used to align the sequences and the alignment was manually corrected. Nucleotide diversity per site (π) was estimated from the *P. vivax* sequences and the modified Nei-Gojobori method [28] was used to assess natural selection signals by calculating the difference between synonymous and non-synonymous substitution rates (d_N_-d_S_). Natural selection was also assessed by estimating the difference between synonymous and non-synonymous divergence rates (K_N_-K_S_) using sequences from *P. vivax* and related species through the modified Nei-Gojobori method and Jukes-Cantor correction [29]. Specific codons under natural selection amongst species were identified using codon-based Bayesian or maximum likelihood approaches (SLAC, FEL, REL [30], MEME [31] and FUBAR [32]), following recombination by the GARD method [33]. Codon-based methods estimate the evolutionary rate (ω) at each codon using a statistical test to determine whether ω is significantly different to 1 (neutral evolution). The Branch-site REL algorithm [34] was used to identify lineages under episodic positive selection (selection occasionally having transient periods of adaptive evolution masked by negative selection or neutral evolution). The Datamonkey web server was used to perform these analyses [35].

### Primer design, cloning and sequencing

**Fig. 1:**
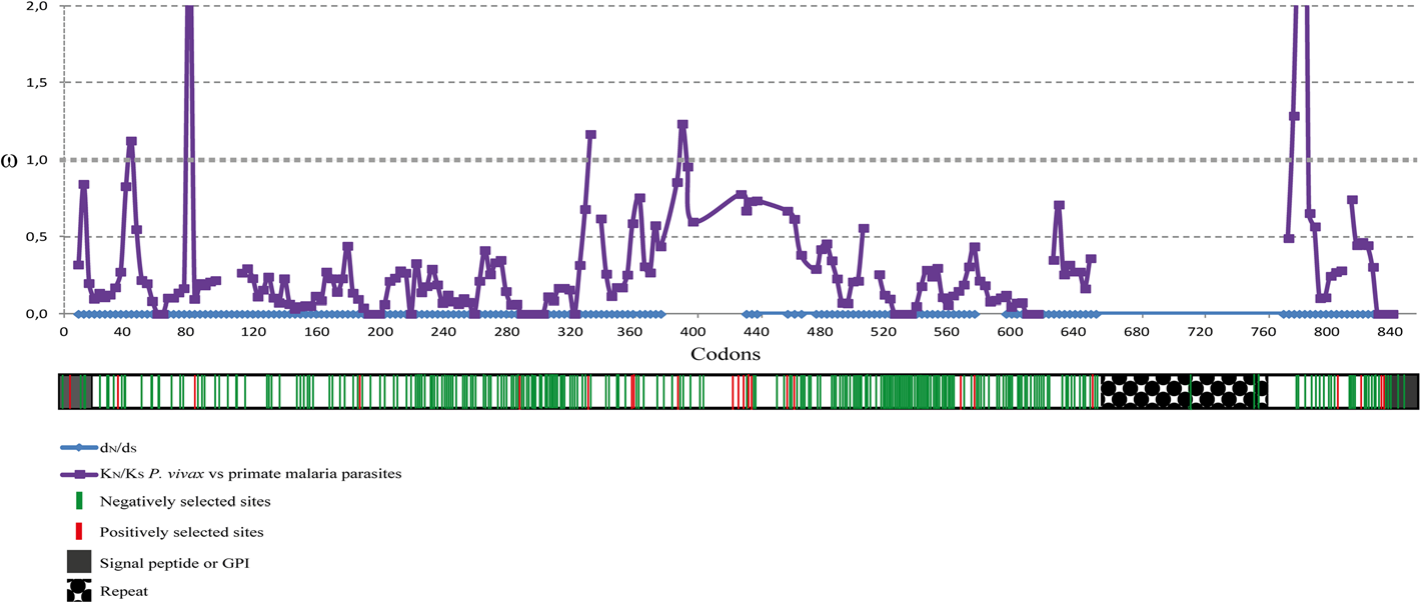
Evolutionary rate (ω) sliding window. Intra-species ω values (d_N_/d_S_) are represented in *blue* whilst inter-species ω values (K_N_/K_S_ between *P. vivax* and malarial parasites infecting primates) are shown in *purple*. A ω value equal 1 means neutral evolution, ω < 1 negative selection whilst ω > 1 means positive selection. A diagram of the gene can be observed *below* the sliding window. Negatively selected inter-species codons are shown in *green* whilst positively selected sites are shown in *red*. Numbering is based on the alignment in Additional file 1: Figure S1

The *Plasmodium vivax gama* (*pvgama*) gene sequence was taken from the PlasmoDB database [36] and scanned for PCR priming sites (Table 1) using Generunner software (version 3.05). Primers were designed to amplify either the entire *pvgama* gene or several smaller-sized fragments according to the natural selection analysis (Fig. 1). The gDNA (extracted using a Wizard Genomic purification kit; Promega, Madison, USA) and cDNA (synthesised with SuperScript III enzyme (RT+) (Invitrogen, Carlsbad, USA) samples from *P. vivax* VCG-I strain schizont-stage enriched parasites (propagated and obtained as previously described [37, 38]) were used as template in 25 μl PCR reactions containing 1× KAPA HiFi HotStart ReadyMix (KAPA Biosystems, Woburn, MA, USA), 0.3 μM primers and DNAse-free water. Temperature cycling for PCR involved a denaturing step of 95 °C for 5 min, followed by 35 cycles of 98 °C for 20 s, Tm °C (Table 1) for 15 s and 72 °C for 30 s or 1 min and 30 s depending on product size. A Wizard PCR preps kit (Promega) was used for purifying amplicons obtained from PCR with the RT+ and gDNA samples, once quality had been evaluated on agarose gel. Purified products were ligated to the pEXP5 CT/TOPO expression vector or pGEM (Promega) (for the gene obtained from gDNA) and transformed in TOP10 *E. coli* cells (Invitrogen). Several clones obtained from independent PCR reactions were grown for purifying the plasmid using an UltraClean mini plasmid prep purification kit (MO BIO Laboratories, California, USA). Insert integrity and correct orientation were then confirmed by sequencing, using an ABI-3730 XL sequencer (MACROGEN, Seoul, South Korea). ClustalW (NPS@) software was used for comparing gene sequences from Sal-I reference strain and the primate-adapted VCG-I strain [39]. The *pvgama* gene sequence from *P. vivax* VCG-I strain was deposited in NCBI under accession number KT248546.

**Table 1:** Primer designed for *pvgama* gene amplification

Target	Primer sequence (5’ – 3’)^a^	MT (°C)	Product size (bp)	aa position
*pvgama*	Fwd: ATGAAGTGCAACGCCTCC Rev: AAAAATGAATAGGAGCAACG	58	2313	1 to 771
*pvgama* -Nt	Fwd: ATACGGAATGGAAACAACC Rev: AGTCGGTTCGTTATTCTCG		1284	22 to 449
*pvgama* -Ct	Fwd: CTGCTCAAGAACACGAAC Rev: GCTTCCACTCTGCAATTC		948	434 to 749
*pvgama* -CR1	Fwd: GACGATCATCTGTGTTCAAAAA Rev: GACCTCATTTTTGGACTTCTC	60	666	87 to 308
*pvgama* -VR1	Fwd: GGCGCCTTCCTGCAGTC Rev: CATTAACATGGTGTTGTCGCT		438	330 to 475
*pvgama* -CR2	Fwd: CAGGCGGCCATCTTACTAA Rev: GCTCCCGTTGACGCCCTT		321	482 to 588
*pvgama* -VR2	Fwd: GCCGCAAACGCAGACGCC Rev: GTTTGCCGAGAAGCTTCCAC		384	626 to 753

*Abbreviations*: *Nt and Ct* amino and carboxyl terminal; *CR* conserved region, *VR* variable region; *Fwd* forward, *Rev* reverse, *MT* melting temperature, *bp* base pair, *aa* amino acid

^**a**^Protein’s expression start codon was included in forward primer’s 5’ end

### Recombinant protein expression

The pEXP-*pvgama* recombinant plasmids were transformed in *E. coli* BL21-DE3 (Invitrogen), according to the manufacturer’s recommendations. Cells were grown overnight at 37 °C in 50 ml Luria Bertani (LB) medium containing 100 μg/ml ampicillin using a Lab-line Incubator Shaker. The initial inoculum was then seeded in 1 l of LB with ampicillin (100 μg/ml) and left to grow at 37 °C with shaking at ~300× *rpm* until reaching 0.5 OD_600_. The culture was incubated on ice for 30 min and then IPTG 1 mM was used to induce expression by incubation for 16 h at room temperature (RT) with shaking at ~200× *rpm*. The culture was then spun at 2400× *g* for 20 min and the pellet was collected for extraction of the recombinant protein.

### Denaturing extraction

The cell pellet obtained from *E. coli* expressing *Pv*GAMA-Nt and *Pv*GAMA-Ct fragments was homogenised in denaturing extraction buffer (DEB) (6 M urea, 10 mM Tris, 100 mM NaH_2_PO_4_ and 20 mM imidazole) containing the SIGMAFAST protease inhibitor cocktail (Sigma-Aldrich, St. Louis, USA) and then lysed by incubating with 0.1 mg/ml lysozyme overnight at 4 °C at 10× *rpm* using a tube rotator (Fisher Scientific, Waltham, USA). The supernatant was collected by spinning at 16,000× *g* for 1 h.

### Native extraction

*Pv*GAMA-CR1, *Pv*GAMA-VR1, *Pv*GAMA-CR2 and *Pv*GAMA-VR2 were extracted using a method for obtaining the molecules in native conditions with the respective positive and negative controls (region II and III/IV from the Duffy binding protein, DBP) (unpublished data). Briefly, the pellet was frozen/thawed for 3 cycles and then homogenised in native extraction buffer (NEB) (50 mM Tris, 300 mM NaCl, 25 mM imidazole, 0.1 mM EGTA and 0.25% Tween-20, pH 8.0). The mixture was incubated for 1 h at 4 °C at 10× *rpm* and the supernatant was collected by spinning at 16,000× *g* for 1 h.

### Protein purification

Total lysate supernatant was incubated with Ni^+2^-NTA resin (Qiagen, Valencia, CA, USA) for purifying the proteins by solid-phase affinity chromatography, once protein expression had been verified by western blot. Briefly, the resin was pre-equilibrated with the respective buffer used for extracting proteins and then incubated with the *E. coli* lysate overnight at 4 °C. The protein-resin mixture was placed on a column and then weakly bound proteins were eluted by washing with 20 ml buffer containing 0.1% Triton X-114 followed by 50 ml of the same buffer without detergent. The proteins extracted in denaturing conditions were dialysed on the column by passing 20 ml DEB with urea in descending concentrations (6 M, 3 M, 1.5 M, 0.75 M and PBS). Bound proteins were then eluted with PBS containing imidazole at increasing concentrations (50 mM to 500 mM) in 3 ml fractions; those having a single band (confirmed on 12% SDS-PAGE by Coomassie blue staining and by western blot using anti-polyhistidine antibodies) were pooled and dialysed extensively in PBS, pH 7.2. A micro BCA protein assay kit (Thermo Scientific, Rockford, USA) was used for quantifying each protein, using the bovine serum albumin (BSA) curve as reference.

### Peptide synthesis

One 6 histidine peptide was synthesised according to a previously-established methodology [40], polymerised, lyophilised and characterised by RP-HPLC and MALDI-TOF MS. The peptide was homogenised in PBS and then stored at -20 °C until use.

### Blood sample collection and processing

Individuals with a clinical history of *P. vivax* (37 subjects) or *P. falciparum* (30 subjects) malaria, aged 18 to 50 year-old and living in malaria-endemic areas of Colombia (Chocó, Nariño, Córdoba, Vichada and Guaviare) were selected for this study. Sera from healthy individuals (16 adult subjects) who had never been affected by the disease and who were living in non-endemic areas were used as negative controls. The blood samples were collected in BD Vacutainer tubes without anticoagulant by personnel from the Fundación Instituto de Inmunología de Colombia (FIDIC) from October 2006 to March 2011 (for *P. vivax*) and June to October 1993 (for *P. falciparum*) and stored at 4 °C until transport. Samples were then transported to Bogotá for processing. Total blood was spun at 5000× *g* for 5 min and the serum was then recovered and stored at -80 °C in FIDIC serum bank (to date).

### Enzyme-linked immunosorbent assay (ELISA)

*Pv*GAMA antigenicity was evaluated in triplicate using serum from patients who had suffered episodes of *P. vivax* or *P. falciparum* infection. Briefly, 96-well polysorb plates were covered with 1 μg r*Pv*GAMA-Nt, or r*Pv*GAMA-Ct, overnight at 4 °C and then incubated at 37 °C for 1 h. The dishes were blocked with 200 μl 5% skimmed milk - PBS-0.05% Tween for 1 h at 37 °C. Antibody reactivity against the recombinant protein was evaluated by incubating the plates with 1:100 dilution of each human serum in 5% skimmed milk - PBS-0.05% Tween for 1 h at 37 °C. The dishes were incubated with peroxidase-coupled goat anti-human IgG monoclonal secondary antibody (1:10,000) (Catalogue 1222H, ICN) diluted in 5% skimmed milk - PBS-0.05% Tween for 1 h at 37 °C and then a peroxidase substrate solution (KPL Laboratories, Gaithersburg, MD, USA) was added to reveal the reaction, according to the manufacturer’s recommendations. Optical density (OD) at 620 nm (detected by MJ ELISA Multiskan Reader) was calculated by subtracting the OD value obtained from the control well value (no antigen). The cut-off value for evaluating the positivity threshold was determined by taking the average of the OD plus twice the standard deviation (± 2SD) of healthy individuals’ sera reactivity.

### Cord blood sample processing

The newborn umbilical cord blood samples used in this research were collected by personnel from the Hemocentro Distrital (Bogotá) and then processed by SEPAX Cell Processing System (Biosafe, Eysins, Switzerland) to reduce nucleated cells, according to the manufacturer’s recommendations. The samples were stored at 4 °C and Duffy antigen receptor for chemokines (DARC) presence was determined by agglutination assay using antibodies directed against the molecule’s Fya or Fyb fraction. The percentage of nucleated cells was scored in 20 fields at 100× magnification using Wright’s stain before carrying out the binding assay.

### Cell binding assay

Reticulocyte binding was tested in triplicate by flow cytometry and using the total cells from cord blood sample (Fya^-^Fyb^+^ phenotype). Briefly, 5 μl samples were incubated with 25 μg of each recombinant protein (*Pv*GAMA-CR1, *Pv*GAMA-VR1, *Pv*GAMA-CR2 and *Pv*GAMA-VR2) for 16 h at 4 °C at 4× *rpm*. Twenty-five μg of DBP region II and III/IV were used as positive and negative controls, respectively. The 6 histidine peptide was also used as control once the recombinant proteins contained a 6-histidine tag. A binding inhibition assay was also performed by incubating *Pv*GAMA conserved recombinant proteins (CR1 and CR2) with a mixture of human sera (1:10 dilution) for 1 h at 4 °C before putting them in contact with cells. The samples were then incubated with mouse anti-His-PE monoclonal antibody (1:40 dilution) (MACSmolecular-Miltenyi Biotec, San Diego, CA, USA) for 30 min in the dark after washing with 1% BSA-PBS solution (v/v). White cells and reticulocytes were stained by incubating with anti-CD45 APC clone 2D1 (1:80 dilution) (Becton Dickinson, Franklin Lakes, NJ, USA) and anti-CD71 APC-H7 clone M-A712 (1:80 dilution) (Becton Dickinson) monoclonal antibodies for 20 min at RT. Subsequently, reticulocyte (CD71 + CD45-PE+) and mature erythrocyte (CD71-CD45-PE+) binding was quantified by analysing 1 million events using a FACSCanto II cytometer (BD, San Diego, CA, USA) and Flowjo V10 software. PE signal intensity in the reticulocyte population was evaluated regarding CD71 signal to determine CD71 low (CD71^lo^) and high (CD71^hi^) cells.

### Statistical analysis

Mean values and standard deviations (SD) were calculated from the measurements of three independent experiments. Statistical significance was assessed by comparing means using a 0.05 significance level for testing a stated hypothesis. Student’s *t*-test and analysis of variance (ANOVA) were used for comparing the means of each experimental group to those for control. Tukey’s multiple comparison test was used for multiple comparison of experimental group means to those for control. GradhPad Software (San Diego, CA) was used for all statistical analysis.

## Results

### *Pv*GAMA genetic diversity and selection signals

*Pvgama* sequences were obtained from genomes of 5 different strains from different geographical regions (North Korea, Brazil, Mauritania and India). These were aligned with the VCG-I strain sequence and orthologous sequences from 5 phylogenetically-related species. The alignment revealed a size polymorphism in *pvgama* due to the [C/T]C[G/C]C[A/T]AA[C/T][C/G][A/G/C][G/A]AC[G/C/A] repeat which was not present in *P. cynomolgi*, *P. inui*, *P. fragile*, *P. knowlesi* or *P. coatneyi* (Additional file 1: Figure S1). Regarding *P. vivax*, 5 segregating sites and π = 0.0008 were observed.

No significant values were found when evaluating synonymous and non-synonymous substitution rates (d_N_-d_S_ = -0.001 (0.001), *P* > 0.1). However, synonymous divergence was greater than non-synonymous divergence (*P* < 0.0001) when comparing *pvgama* sequences to each related species: K_N_-K_S_ *P. vivax/P. cynomolgi* = -0.041 (0.006); K_N_-K_S_ *P. vivax/P. inui* = -0.062 (0.008); K_N_-K_S_ *P. vivax/P. fragile* = -0.030 (0.006); K_N_-K_S_ *P. vivax/P. knowlesi* = -0.072 (0.009); K_N_-K_S_ *P. vivax/P. coatneyi* = -0.049 (0.007). The evolutionary rate ω (dN/dS and KN/KS) sliding window showed that two highly conserved regions amongst species (codons 80–320 and 514–624) might be under negative selection (ω < 0.5). Furthermore, 308 negatively-selected codons were observed amongst species (Fig. 1); a lot of them were in the conserved regions. The Branch-site REL algorithm identified episodic positive selection signals in the lineages giving rise to *P. knowlesi* and *P. coatneyi* as well as the lineage formed by *P. cynomolgi* and *P. fragile* (Additional file 2: Figure S2). 22 sites showed evidence of positive selection amongst species (Fig. 1).

### Antigenic response was directed against the GAMA carboxyl fragment

**Fig. 2:**
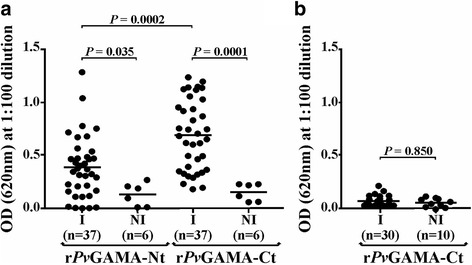
*Pv*GAMA antigenicity during natural malaria infection. The *dot plot* shows OD distribution (Y-axis) for detecting r*Pv*GAMA-Nt or r*Pv*GAMA-Ct by *P. vivax* (**a**) or r*Pv*GAMA-CT by *P. falciparum* (**b)** infected (I) and non-infected (NI) patients’ sera (X-axis). r*Pv*GAMA-Nt: infected individuals *n* = 37, *m* ± SD = 0.38 ± 0.29; control individuals *n* = 6, *m* ± SD = 0.12 ± 0.1. r*Pv*GAMA-Ct: infected individuals *n* = 37, *m* ± SD = 0.67 ± 0.32; control individuals *n* = 6, *m* ± SD = 0.14 ± 0.08. r*Pv*GAMA-Ct recognised by *P. falciparum* infected patients’ sera: infected individuals *n* = 30, *m* ± SD = 0.06 ± 0.04; control individuals *n* = 10, *m* ± SD = 0.06 ± 0.03

Based on the polymorphism analysis results, it was hypothesised that the carboxyl region was more antigenic than the amino one by the presence of the repetitive region. Hence, r*Pv*GAMA-Nt and r*Pv*GAMA-Ct antigenicity (obtained recombinantly; Additional file 3: Figure S3a, b) was evaluated using sera from 37 patients suffering of *P. vivax* malaria and sera from people who had never suffered the disease. r*Pv*GAMA-Nt reacted positively with 64.8% of the sera in screening (0.26 cut-off point) whilst 67.5% of them recognised r*Pv*GAMA-Ct (0.47 cut-off point). These data agreed with a study of the profile of the humoral immune response for *P. vivax* in which r*Pv*GAMA was recognised by 54.5% of the sera used in the array [41]. The statistical test for the assay with r*Pv*GAMA-Nt gave a significant difference between the means (*m*) of the groups (ANOVA: *F*_(1,41)_ = 4.73, *P* = 0.035; *m* = 0.38 for the group of infected patients and *m* = 0.12 for the control group). Likewise, there was a significant difference between the means of the groups (ANOVA: *F*_(1,41)_ = 14.75, *P =* 0.0001; *m* = 0.67 for the group of infected patients and *m* = 0.14 for the control group) when r*Pv*GAMA-Ct was detected by human sera (Fig. 2a). There was also a statistically significant difference when analysing the means of recognition for r*Pv*GAMA-Nt and r*Pv*GAMA-Ct (ANOVA: *F*_(1,72)_ = 16.01, *P =* 0.0002). Taking into account that the response was higher against *Pv*GAMA-Ct, it was decided to confirm whether the antibodies generated during *P. falciparum* natural infection were able to detect this fragment. No significant difference (ANOVA: *F*_(1,38)_ = 0.036, *P =* 0.850) was seen for *Pv*GAMA-Ct recognition by these sera (Fig. 2b). The significant reactivity against the recombinants by *P. vivax*-infected individuals’ sera indicated that the protein could trigger an antigenic response during natural infection, this being higher and species-specific against the *Pv*GAMA carboxyl region.

### *Pv*GAMA bound to human reticulocytes

**Fig. 3:**
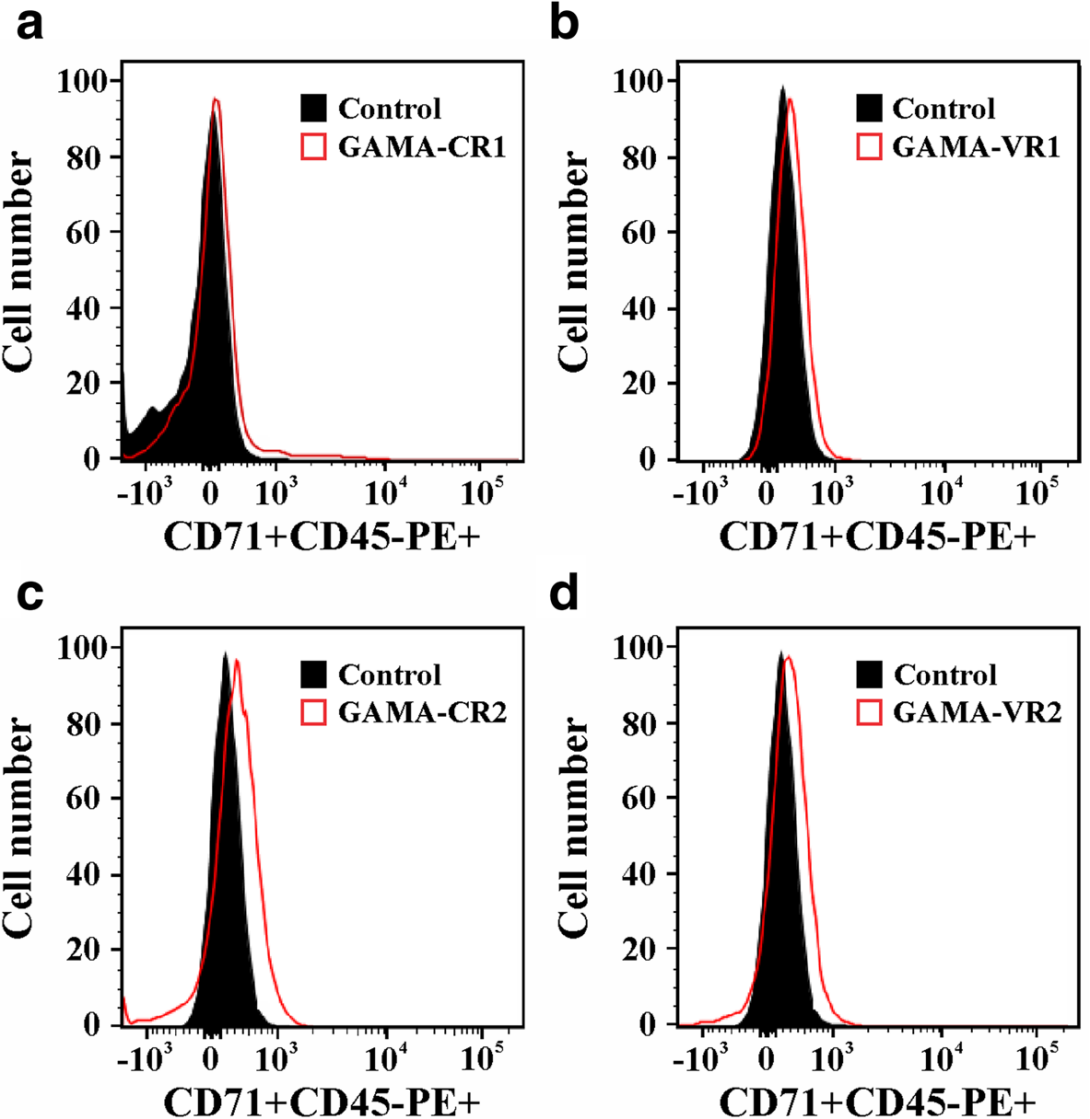
Flow cytometry analysis. Histograms of conserved (**a** and **c**) and variable (**b** and **d**) GAMA fragments compared to control (cells not incubated with the protein). Each figure is representative from three independent experiments

**Fig. 4:**
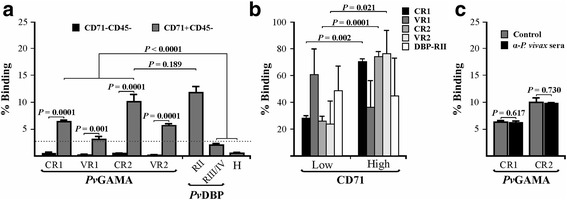
*Pv*GAMA human reticulocyte binding activity. Flow cytometry analysis showing the recombinant binding percentage to CD71-CD45- and CD71 + CD45- cells (**a**) and regarding CD71-APCH7 signal (only for CD71 + CD45- cells) (**b**). Positive (DBP-RII) and negative (DBP-RIII/IV and H (6 histidine peptide)) binding controls are also shown. **c** CR1 and CR2 reticulocyte binding inhibition assay using human sera (α-*P. vivax* sera). Binding percentage in both analyses were expressed as mean ± SD of three independent experiments

Red blood cell samples having the Fya^-^Fyb^+^ phenotype (Duffy +) taken from umbilical cord blood were incubated with conserved (CR1 and CR2) and variable (VR1 and VR2) regions extracted and purified in their soluble form (Additional file 3: Figure S3c), predicted by natural selection analysis and then evaluated by flow cytometry to quantify the protein-cell interaction. The percentage of each recombinant binding to erythrocytes was calculated using the gating strategy described in Additional file 4: Figure S4, which enabled selecting the mature (CD71-CD45-) or immature (CD71 + CD45-) cell population to which a target protein was bound (labelled with anti-His PE antibody). All recombinant proteins had a curve shift when the PE signal was compared to control (cells not incubated with recombinant proteins) in the histogram (Fig. 3). Interestingly, the GAMA fragments bound to reticulocytes to a much higher percentage compared to mature erythrocytes (CR1: *t*-test: *t*_(4)_ = 24.9, *P* = 0.0001; VR1: *t*-test: *t*_(4)_ = 9.02, *P* = 0.001; CR2: *t*-test: *t*_(4)_ = 12.4, *P* = 0.0001; VR2: *t*-test: *t*_(4)_ = 24.8, *P* = 0.0001) (Fig. 4a). The conserved regions showed highest interaction with the reticulocytes compared to negative binding controls (ANOVA-Tukey: *F*_(6, 12)_ = 72.64, *P* < 0.0001). CR2 recombinant protein bound to 10.11% (SD = 1.33) of target cells, which was very similar to the positive control (*m* ± SD = 11.8 ± 1.15) (*P* > 0.189), whilst CR1 were able to bind to 6.36% (SD = 0.30) of the cells (Fig. 4a). Regarding *Pv*GAMA variable regions, VR1 was able to bind to 3.08% (SD = 0.54) of the reticulocytes whilst VR2 bound 5.64% (SD = 0.37). CR1, CR2 and VR2 fragments had the highest interaction with CD71^hi^ reticulocytes when binding percentages were analysed as a function of CD71 APC-H7 signal (CR1: *t*-test: *t*_(4)_ = 7.32, *P* = 0.002; CR2: *t*-test: *t*_(4)_ = 16.04, *P* = 0.0001; VR2: *t*-test: *t*_(4)_ = 3.71, *P* = 0.021), unlike VR1 and DBP-RII (VR1: *t*-test: *t*_(4)_ = 1.52, *P* = 0.202; DBP-RII: *t*-test: *t*_(4)_ = 0.19, *P* = 0.853) (as previously found [42]) (Fig. 4b). These findings suggested that GAMA in *P. vivax* has a functional role in preferential interaction with human reticulocytes.

### Natural antibodies did not affect *Pv*GAMA binding activity

A cytometry adhesion inhibition assay was performed with sera from individuals suffering *P. vivax* malaria to determine whether the antibodies produced during natural infection could inhibit functional conserved regions (CR1 and CR2) interaction with reticulocytes. Figure 4c shows that conserved recombinant proteins pre-incubated with human sera were able to bind to target cells (CR1: *m* ± SD = 6.21 ± 0.27; CR2: *m* ± SD = 9.83 ± 0.09), giving a similar percentage to that for controls (CR1: *m* ± SD = 6.5 ± 0.08; CR2: *m* ± SD = 10.01 ± 0.95) (CR1: *t*-test: *t*_(3)_ = 0.55, *P* = 0.617; CR2: *t*-test: *t*_(4)_ = 0.37, *P* = 0.730), suggesting that the immune response was directed against regions which are not implicated in cell binding.

## Discussion

Merozoite invasion of erythrocytes involves the participation of several parasite molecules expressed at the end of the intra-erythrocyte lifecycle, mainly those contained in the apical organelles, such as the rhoptries and micronemes [8]. Only a few of these molecules possessing a reticulocyte binding role in *P. vivax* have been identified and their binding domains mapped, suggesting an urgent need for performing further studies to supplement current knowledge on *P. vivax* adhesins. This will improve our understanding of the molecular basis of parasite invasion of reticulocytes. This study aimed at using natural selection analysis for identifying GAMA functional regions playing a potential role in reticulocyte binding.

According to the phylogenetic analysis, a repeat region (RR) localised between amino acids 591 and 695 consisting of residues [A/L]AN[A/G][N/D] was predicted. This RR was common in different *P. vivax* strains but not in phylogenetically-related species (Additional file 1: Figure S1). This characteristic has been found in several *P. vivax* antigens described in the *P. vivax* VCG-I strain located on the parasite surface (Pv12 [12], ARP [43]) or in the apical pole (Pv34 [44], RON1 [45], RON2 [46] and RON4 [47, 48]). DNA sequences from different *P. vivax* strains and phylogenetically-related species were thus compared to ascertain whether *gama* gene diversity has been modulated by immune pressure. Evidence of episodic positive selection was found in some parasite lineages (Additional file 2: Figure S2). As shown for other antigens [49–51], the episodic selection found in GAMA could be the outcome of adaptation to different hosts during malaria-primate evolution [50, 51]. Therefore, the insertions found in *P. vivax* could be an adaptation of the species to humans since the RR in malaria are associated with evasion of the host’s immune response, making such response become directed against functionally unimportant regions [52, 53]. This hypothesis was supported by the fact that r*Pv*GAMA-Ct (where the RR is located) can trigger a species-specific immune response (Fig. 2) which did not inhibit CR2 binding activity to reticulocytes (Fig. 4c).

Polymorphic regions induce high levels of strain-specific antibodies (allele specific) whilst conserved regions (directly implicated in interaction with cell receptors) are usually non-antigenic [54]. Therefore, the immune response must be directed against conserved regions to avoid different parasite strains evading immunity, thereby reducing vaccine efficacy. According to the selection signal identification strategy, low genetic diversity was found in the GAMA-encoding gene, comparable to that observed in *msp4* [55, 56], *msp7A/7 K/7 F/7 L* [57, 58], *msp8* [59], *msp10* [57, 59], *pv12*, *pv38* [22, 24], *pv41* [23, 24], *rap1/2* [60] and *ron4* [48] which seem involved in host cell invasion. Despite the lack of statistically significant values for d_N_-d_S_ difference, K_S_ divergence amongst species was greater than K_N_, suggesting negative selection. Many codons were found to be experiencing negative selection which probably plays an important role in GAMA evolution. Two regions along the antigen were highly conserved amongst species, giving a < 0.5 evolutionary rate (ω) (Fig. 1).

Given the polymorphism and selection analysis, it was decided to determine *Pv*GAMA conserved and variable region interaction with reticulocytes to validate the *in silico* prediction of functional regions (Figs. 3 and 4) and elucidate the protein’s function. A reticulocyte sample having a Duffy positive phenotype was used, given that *Pv*GAMA reportedly has a binding role regardless of such antigen’s expression [61]. Unlike Cheng and his group, the anti-CD71 monoclonal antibody was included for identifying GAMA regions’ preference for immature reticulocyte binding as *P. vivax* merozoites have tropism for this cell type (characterised by the expression of the CD71 receptor [62]). Given that the CD71 marker is also present in activated lymphocytes, a nucleated cell depleted umbilical cord blood sample was used. The anti-CD45 was also included to totally exclude the lymphocytes from the analysis once the Wright staining revealed 0.4% of such cells (also confirmed by cytometry analysis) (Additional file 4: Figure S4). It was also confirmed that there was no difference in reticulocyte percentage by incubating the samples for 4 and 16 h at 4 °C (4 h: *m* ± SD = 1.24 ± 0.27; 16 h: *m* ± SD = 1.31 ± 0.07) (*t*-test: *t*_(2)_ = 0.32, *P* > 0.777). However, it was decided to use a prolonged incubation time to enable complete protein-cell interaction.

It was found that all *Pv*GAMA fragments bound to mature erythrocytes (CD71-CD45-) though to a lesser extent compared to reticulocytes (CD71 + CD45-) (Fig. 4a), thereby supporting the fact that the protein preferentially interacts with the latter cell type. The conserved fragment located in the carboxyl region (CR2) had higher reticulocyte binding than the amino one (CR1) (Fig. 4a) coinciding with that shown recently for *Pv*GAMA where this fragment [F2 (aa 345 to 589) or F7 (408 to 589) regions in that study] showed higher rosetting activity, unlike the F1 region (aa 22 to 344) (amino fragment) [61]. Interestingly, CR1 and CR2 had higher CD71^hi^ reticulocyte binding percentages than to CD71^lo^ (Fig. 4b), suggesting that GAMA mainly binds to such cell type’s most immature stage. It has been reported that some reticulocytes’ integral membrane components decrease as cells mature [63]. Therefore, the findings found here suggest that *Pv*GAMA receptor is less abundant in CD71^lo^ cells unlike CD71^hi^, as a consequence of cell maturation. The fact that more than 69% of the CD71 + CD45- cells were CD71^lo^ (*m* ± SD = 69.3 ± 3.3) can be the explanation of why *Pv*GAMA fragment binding to 100% of the CD71+ reticulocytes was not found (Fig. 4a). It has been observed that several *P. vivax* proteins, such as DBP [64], MSP-1 [16], RBP1 [14], the erythrocyte binding protein (EBP) [42], RBP1a, RBP1b [65] and RBP2 [15], have preferential reticulocyte binding activity, being the RBPs particularly important in parasite cell selection. Taking the results obtained here into account, it can be suggested that *P. vivax* target cell selection is not only governed by the RBPs but other ligands are also taking place in this process, such as DBP, MSP-1, EBP and now *Pv*GAMA.

Immunoreactive proteins are considered potential candidates for developing a vaccine as it has been seen that an immune response induced during infection is related to naturally-acquired immunity [66]. Antigenicity is thus one of the classical parameters for selecting molecules when developing a vaccine. Although there was an immune response against *Pv*GAMA (Fig. 2), this was not sufficient to inhibit the conserved regions binding to reticulocytes (Fig. 4c). It has been observed that *P. falciparum* proteins’ conserved regions (implicated in target cell binding) cannot trigger an immune response when used as vaccine candidates in the *Aotus* model whilst non-conserved ones trigger protective responses upon parasite challenge but those are strain-specific [54]. Accordingly, the *Pv*GAMA antibodies produced/induced during natural *P. vivax* infection were directed against immunodominant epitopes which are unimportant in binding activity. Bearing in mind that functional regions usually evolve more slowly and that natural negative selection tends to keep these regions conserved amongst species [25], our experimental findings suggested that CR1 and CR2 located between residues 80–320 (40% of negatively selected sites) and 514–624 (64.5% of negatively selected sites) are functionally/structurally restricted and that vaccine design should thus be focused on them.

## Conclusions

To our knowledge, this study described *Pv*GAMA reticulocyte binding properties for the first time. The *Pv*GAMA antigenic response was principally directed against its carboxyl fragment which comprises by a repetitive region. On the other hand, it was shown that *Pv*GAMA consists of two conserved binding fragments that bind preferentially to most immature human reticulocytes, which is consistent with the *P. vivax* invasion phenotype and highlights the fact that functional regions can be predicted by analysing natural selection. Further studies aimed at discerning the function of conserved regions as vaccine components are required.

## Additional files

Additional file 1: Figure S1. GAMA antigen alignment. *pvgama* sequences from 6 *P. vivax* strains were aligned with orthologous sequences from *P. cynomolgi*, *P. inui*, *P. fragile*, *P. coatneyi* and *P. knowlesi*. a DNA sequence alignment. b Deduced amino acid alignment. The sequences were obtained from GenBank: access numbers being India-VII AFBK01000586-AFBK01000587, North Korean AFNJ01000531, Brazil-I AFMK01000508-AFMK01000509, Mauritania-I AFNI01000333-AFNI01000334, *P. inui* NW_0084818881, *P. fragile* NW_012192586, *P. cynomolgi* BAEJ01000249, *P. coatneyi* CM0028561 and *P. knowlesi* NC_0119061. (PDF 373 kb)

Additional file 2: Figure S2. Lineage-specific positive selection. Branches under positive episodic selection were identified by using the REL-site branch method. Episodic selection acts very quickly and involves a switch from negative to positive natural selection and back to negative and might enable adaptation to a new host. Phylogeny was inferred in MEGA v6 by the maximum likelihood method using the GTR + G evolutionary model. ω^+^ model: ω rate values. Pr [ω = ω +]: percentage of sites evolving under positive selection. *P*-value corrected for multiple tests using the Holm-Bonferroni method. (TIF 470 kb)

Additional file 3: Figure S3. Obtaining recombinant proteins. a, b Recombinant GAMA protein expression and purification. Lanes 2–3 show non-induced and induced cell lysate, respectively. Lanes 4–5 show purified r*Pv*GAMA-Nt and -Ct stained with Coomassie blue or analysed by western blot using anti-polyhistidine antibodies, respectively. **c** Purifying conserved (CR1 and CR2) and variable (VR1 and VR2) *Pv*GAMA regions. Lanes 2, 4, 6 and 8 show purified recombinant proteins and lanes 3, 5, 7 and 9 show western blot detection. The proteins’ molecular markers are indicated in Lane 1 on all figures. (TIF 5327 kb)

Additional file 4: Figure S4. Selection strategy for immature and mature erythrocyte populations. The doublets were excluded by plotting FSC-H against FSC-A. Cells were then selected by their granularity, using an SSC-A vs FSC-A cytogram. The CD45 vs CD71 signal was plotted for selecting reticulocyte (CD71 + CD45-) and mature erythrocyte (CD71-CD45-) populations and omitting activated lymphocytes (CD71 + CD45+). The percentage of cells having bound protein was calculated using the PE signal (CD71 + CD45-PE+). A representative histogram from three independent experiments analysing the PE signal for the CR2 binding assay compared to control is also shown. (TIF 10448 kb)

## Abbreviations

ANOVA: Analysis of variance
CD71^hi^: CD71 high
CD71^lo^: CD71 low
CR: Conserved region
DARC: Duffy antigen receptor for chemokines
DBP: Duffy binding protein
DEB: Denaturing extraction buffer
EBP: Erythrocyte binding protein
ELISA: Enzyme-linked immunosorbent assay
LB: Luria bertani
MALDI-TOF: Matrix-assisted laser desorption/ionization-time of flight
MS: Mass spectrometry
MSP-1: Merozoite surface protein 1
NEB: Native extraction buffer
OD: Optical density
PBS: Phosphate buffered saline
*Pv*GAMA: *P. vivax* GPI-anchored micronemal antigen
*Pvgama*: *Plasmodium vivax gama*
RBP: Reticulocyte binding protein
RON5: Rhoptry neck protein 5
RP-HPLC: Reverse phase high-performance liquid chromatography
RR: Repeat region
RT: Room temperature
SD: Standard deviation
TRAg: Tryptophan-rich antigen
VCG-I: Vivax Colombia Guaviare 1
VR: Variable region
