# Supplementary material for: PvGAMA reticulocyte binding activity: predicting conserved functional regions by natural selection analysis
Source: Parasit Vectors. 2017 May 19;10:251. doi: 10.1186/s13071-017-2183-8 (PMC5438544; doi:10.1186/s13071-017-2183-8)
Supplement: Supplementary file 1 — GAMA antigen alignment. pvgama sequences from 6 P. vivax strains were aligned with orthologous sequences from P. cynomolgi, P. inui, P. fragile, P. coatneyi and P. knowlesi. a DNA sequence alignment. b Deduced amino acid alignment. The sequences were obtained from GenBank: access numbers being India-VII AFBK01000586-AFBK01000587, North Korean AFNJ01000531, Brazil-I AFMK01000508-AFMK01000509, Mauritania-I AFNI01000333-AFNI01000334, P. inui NW_0084818881, P. fragile NW_012192586, P. cynomolgi BAEJ01000249, P. coatneyi CM0028561 and P. knowlesi NC_0119061. (PDF 373 kb) [file 13071_2017_2183_MOESM1_ESM.pdf]

a. DNA sequence alignment.

```
                                               *        20         *        40         *        60         *        80
P.coatneyi_CM002856.1               : ATGAAGTGCAACGGCTCCTTGCTAGTCCTACTTAGCGCAATACTCAGCGCTACGAACGCTTTGATACGAAATGGGAACAA :   80
Pknowlesi_NC_011906.1               : ATGAAGTGCAACGGCTCCTTGCTAGTCCTACTTAGCGCAATACTCAGCGCCACGAACGCTTTGATACGAAATGGGAACAA :   80
Pinui_NW_008481888.1                : ATGAAGTGCAACACGGCCTTGCTAGTACTACTTAGCGCAATACTCAGCGCTGCAAACGCTTTGATACGAAATGGGAACAC :   80
Pfragile_NW_012192586.1             : ATGAAGTATAACGCCACCTTGCTTGTCCTACTTAGCGCAATATTGAGCGCTGGGAAAGCTTTGATACGAAATGGGAACAA :   80
Pcynomolgi_BAEJ01000249             : ATGAAGTGCAACGCCGCCTTGCTAGTCCTACTTAGCGCATTACTCAGCGCTTCGAACGCTTTGATACGAAATGGGAACAA :   80
Brazil-I_AFMK01000508.1_A           : ATGAAGTGCAACGCCTCCTTGCTAGTCCTACTTAGCGCACTACTCAGCGCTGCGAACGCTTTGATACGGAATGGAAACAA :   80
VCG-I                               : ATGAAGTGCAACGCCTCCTTGCTAGTCCTACTTAGCGCACTACTCAGCGCTGCGAACGCTTTGATACGGAATGGAAACAA :   80
Sal-1_PVX_088910                    : ATGAAGTGCAACGCCTCCTTGCTAGTCCTACTTAGCGCACTACTCAGCGCTGCGAACGCTTTGATACGGAATGGAAACAA :   80
Mauritania_I_AFNI01000333           : ATGAAGTGCAACGCCTCCTTGCTAGTCCTACTTAGCGCACTACTCAGCGCTGCGAACGCTTTGATACGGAATGGAAACAA :   80
NKorean_AFNJ01000531                : ATGAAGTGCAACGCCTCCTTGCTAGTCCTACTTAGCGCACTACTCAGCGCTGCGAACGCTTTGATACGGAATGGAAACAA :   80
India-VII_AFBK01000586_AFBK01000587 : ATGAAGTGCAACGCCTCCTTGCTAGTCCTACTTAGCGCACTACTCAGCGCTGCGAACGCTTTGATACGGAATGGAAACAA :   80
                                      ATGAAGTgcAACgcc CCTTGCTaGTcCTACTTAGCGCA TAcTcAGCGCt cgAAcGCTTTGATACG AATGG AACAa

                                               *       100         *       120         *       140         *       160
P.coatneyi_CM002856.1               : TCCGCAGGCATTAGTTCCGGAAAAGAGCGATGACTCGAGTGGGGGCCAGAACAAACCCTCCGGTGACAACCAAGACACGT :  160
Pknowlesi_NC_011906.1               : TCCGCAGGCATTAGTTCCGGACAATAGTGGTGACCCGAATGCGGGCCAGAACAAAACGTCCAGTGACAACCAAGACACAT :  160
Pinui_NW_008481888.1                : TACGCAGGCATTAGTTCCGGAAAAGAGCAATGACCCGAGTGGGGGCCAGAACAACCCCTCCGGTGACAACCGAGAGACTT :  160
Pfragile_NW_012192586.1             : TACGCAGGCCTTAGTTCCTGAAAATGGCGGTGACCCTAGTGGGGACAGAACAACCCCTCCGGTGAAAACCAAGATACGT :  160
Pcynomolgi_BAEJ01000249             : TACGCAAGCATTAGTTCCGGAAAAGAGCGGTGACCCGAGTGGGGGCCAGAACAACCCCTCCGGTGATAACCAAGACACGT :  160
Brazil-I_AFMK01000508.1_A           : CCCGCAGGCATTAGTTCCTGAAAAGGGCGCTGACCCGAGTGGGGGCCAGAACAACCGCTCCGGAGAAAACCAAGACACGT :  160
VCG-I                               : CCCGCAGGCATTAGTTCCTGAAAAGGGCGCTGACCCGAGTGGGGGCCAGAACAACCGCTCCGGAGAAAACCAAGACACGT :  160
Sal-1_PVX_088910                    : CCCGCAGGCATTAGTTCCTGAAAAGGGCGCTGACCCGAGTGGGGGCCAGAACAACCGCTCCGGAGAAAACCAAGACACGT :  160
Mauritania_I_AFNI01000333           : CCCGCAGGCATTAGTTCCTGAAAAGGGCGCTGACCCGAGTGGGGGCCAGAACAACCGCTCCGGAGAAAACCAAGACACGT :  160
NKorean_AFNJ01000531                : CCCGCAGGCATTAGTTCCTGAAAAGGGCGCTGACCCGAGTGGGGGCCAGAACAACCGCTCCGGAGAAAACCAAGACACGT :  160
India-VII_AFBK01000586_AFBK01000587 : CCCGCAGGCATTAGTTCCTGAAAAGGGCGCTGACCCGAGTGGGGGCCAGAACAACCGCTCCGGAGAAAACCAAGACACGT :  160
                                        CGCAgGCaTTAGTTCC GAaAAg Gcg TGACcCgAgTGgGGGcCAGAACAAcc cTCCgG GA AACCaAGAcACgT

                                               *       180         *       200         *       220         *       240
P.coatneyi_CM002856.1               : GCGAAATTCAAAAGATGGCGGAAGAAATGATGGAAAAAATGGTGAACGAAAAAGATGTGTTTACCTCCATCATGGAACCT :  240
Pknowlesi_NC_011906.1               : GTGAAATTCAAAAGATGGCCGAAGAAATGATGGAAAAAATGATGAAAGAAAAAGACGTGTTTAGCTCCATTATGGAACCT :  240
Pinui_NW_008481888.1                : GTGAAATTCAACAGATGGCCCAAGAAATGATGGAAAAAATGATGAAAGAAAAAGACGTGTTTAGCTCCATCATGGAACCC :  240
Pfragile_NW_012192586.1             : GCGAAGTTCAAAAGATGGCTGAAGAAATGATGGGAAAAATGATGAAAGAAAAAGACGTGTTTAGCTCCATTATGGAACCT :  240
Pcynomolgi_BAEJ01000249             : GTGAAATTCAAAAGATGGCCGAAGAAATGATGGAAAAAATGATGAAAGAAAAAGACATGTTTAGCTCCATCATGGAACCT :  240
Brazil-I_AFMK01000508.1_A           : GCGAAATTCAAAAGATGGCCGAAGAAATGATGGAAAAAATGATGAAGGAAAAAGACGTGTTTAGCTCCATCATGGAACCT :  240
VCG-I                               : GCGAAATTCAAAAGATGGCCGAAGAAATGATGGAAAAAATGATGAAGGAAAAAGACGTGTTTAGCTCCATCATGGAACCT :  240
Sal-1_PVX_088910                    : GCGAAATTCAAAAGATGGCCGAAGAAATGATGGAAAAAATGATGAAGGAAAAAGACGTGTTTAGCTCCATCATGGAACCT :  240
Mauritania_I_AFNI01000333           : GCGAAATTCAAAAGATGGCCGAAGAAATGATGGAAAAAATGATGAAGGAAAAAGACGTGTTTAGCTCCATCATGGAACCT :  240
NKorean_AFNJ01000531                : GCGAAATTCAAAAGATGGCCGAAGAAATGATGGAAAAAATGATGAAGGAAAAAGACGTGTTTAGCTCCATCATGGAACCT :  240
India-VII_AFBK01000586_AFBK01000587 : GCGAAATTCAAAAGATGGCCGAAGAAATGATGGAAAAAATGATGAAGGAAAAAGACGTGTTTAGCTCCATCATGGAACCT :  240
                                      G GAAaTTCAAaAGATGGCcgAAGAAATGATGGaAAAAATGaTGAA GAAAAAGAcgTGTTTAgCTCCATcATGGAACCt
```

```
                                                  *       260         *       280         *       300         *       320
P.coatneyi_CM002856.1                          : CTCCAGAGCAAATTAACCGATGATCACCTCTGTTCAAAACTAAAATATACGAACGTTTGTCTTCACGAAAAGGACAAAAC :  320
Pknowlesi_NC_011906.1                          : CTCCAGAGCAAATTAACCGATGATCGCCTCTGTTCTAAAATGAAATATACAAATATTTGTCTTCACGAAAAGGACAAAAC :  320
Pinui_NW_008481888.1                           : CTCCAGAGCAAATTAGCCGACGATCACCTCTGTTCAGAAGTAAAATATAAGAACATTTGTCTTCAAGAAAAGGACAATAA :  320
Pfragile_NW_012192586.1                        : CTCCAGAGCAAATTATCCGACAATCATCTCTGTTCAACAGTGAAATATACGAACATTTGTCTTCACGAAAAGGATAAAAC :  320
Pcynomolgi_BAEJ01000249                        : CTCCAGAGCAAATTACCCGACGATCACCTCTGTTCAAAACTGAAATATACGAACATTTGTCTTCAGGAAAAGGACAAAAC :  320
Brazil-I_AFMK01000508.1_A                      : CTCCAGAGCAAATTAACCGACGATCATCTGTGTTCAAAAATGAAATATACGAACATTTGTCTTCACGAAAAGGACAAAAC :  320
VCG-I                                          : CTCCAGAGCAAATTAACCGACGATCATCTGTGTTCAAAAATGAAATATACGAACATTTGTCTTCACGAAAAGGACAAAAC :  320
Sal-1_PVX_088910                               : CTCCAGAGCAAATTAACTGACGATCATCTGTGTTCAAAAATGAAATATACGAACATTTGTCTTCACGAAAAGGACAAAAC :  320
Mauritania_I_AFNI01000333                      : CTCCAGAGCAAATTAACCGACGATCATCTGTGTTCAAAAATGAAATATACGAACATTTGTCTTCACGAAAAGGACAAAAC :  320
NKorean_AFNJ01000531                           : CTCCAGAGCAAATTAACCGACGATCATCTGTGTTCAAAAATGAAATATACGAACATTTGTCTTCACGAAAAGGACAAAAC :  320
India-VII_AFBK01000586_AFBK01000587            : CTCCAGAGCAAATTAACCGACGATCATCTGTGTTCAAAAATGAAATATACGAACATTTGTCTTCACGAAAAGGACAAAAC :  320
                                                 CTCCAGAGCAAATTA CcGAcgATCa CT TGTTCaaaA TgAAATATAcgAAcaTTTGTCTTCAcGAAAAGGAcAAaAc

                                                  *       340         *       360         *       380         *       400
P.coatneyi_CM002856.1                          : TCCCTTGACCTTCCCCTGCACAAGTCCGGAGTACGAAAAATTAATTCAGCAATTTACTTATCAAAAGTTATGCAACTCCA :  400
Pknowlesi_NC_011906.1                          : TCCCTTGACCTTCCCATGCACAAATCCACAATACGAACAATTAATTCAGCAATTTACTTATAAAAAGTTATGCAACTCCA :  400
Pinui_NW_008481888.1                           : TTCCTTGACCTTCCCCTGCACAAGTCCGGAGTACGAACAATTAATTCACGAATTTACTTATAAAAAGTTATGCAATTCCA :  400
Pfragile_NW_012192586.1                        : TCCCTTGACCTTCCCCTGTACAAGTCCGCAGTACGAACAATTAATTCACCACTTTACTTATAAAAAGTTGTGTAACTCTC :  400
Pcynomolgi_BAEJ01000249                        : TCCCTTGACCTTACCCTGCACAAGTCCGCAGTACGAACAATTAATTCAACACTTTACTTATAAAAAGTTATGCAACTCCC :  400
Brazil-I_AFMK01000508.1_A                      : TCCCTTGACCTTCCCCTGCACAAGTCCGCAGTACGAACAGCTAATTCATCGCTTCACTTATAAAAAGTTGTGCAACTCCA :  400
VCG-I                                          : TCCCTTGACCTTCCCCTGCACAAGTCCGCAGTACGAACAGCTAATTCATCGCTTCACTTATAAAAAGTTGTGCAACTCCA :  400
Sal-1_PVX_088910                               : TCCCTTGACCTTCCCCTGCACAAGTCCGCAGTACGAACAGCTAATTCATCGCTTCACTTATAAAAAGTTGTGCAACTCCA :  400
Mauritania_I_AFNI01000333                      : TCCCTTGACCTTCCCCTGCACAAGTCCGCAGTACGAACAGCTAATTCATCGCTTCACTTATAAAAAGTTGTGCAACTCCA :  400
NKorean_AFNJ01000531                           : TCCCTTGACCTTCCCCTGCACAAGTCCGCAGTACGAACAGCTAATTCATCGCTTCACTTATAAAAAGTTGTGCAACTCCA :  400
India-VII_AFBK01000586_AFBK01000587            : TCCCTTGACCTTCCCCTGCACAAGTCCGCAGTACGAACAGCTAATTCATCGCTTCACTTATAAAAAGTTGTGCAACTCCA :  400
                                                 TcCCTTGACCTTcCCcTGcACAAgTCCgcAgTACGAAcA  TAATTCA c  TT ACTTATaAAAAGTT TGcAAcTCca

                                                  *       420         *       440         *       460         *       480
P.coatneyi_CM002856.1                          : AGGTGGCCTTCAGCAACGTGTTGCTGAAATCCTTCATCGATAAGAAGAACGAAGAAAACACATTCAACGCGATCATACAG :  480
Pknowlesi_NC_011906.1                          : AGGTGGCCTTCAGCAACGTGTTGCTGAAATCCTTCATCGATAAAAAGAACGAAGAAAACACATTTAACGCGATCATACAG :  480
Pinui_NW_008481888.1                           : AGGTGGCCTTTAGCAACGTGTTGCTCAAATCGTTCATCGATAAAAAAAACGAAGAAAACACATTTAACACGATCATAGAG :  480
Pfragile_NW_012192586.1                        : AAGTGGCCTTTAACAACGTGTTGCTCAAATCCTTCATCAATAAAAACAACGAAGAAAACACATTTAACACGATTATACAG :  480
Pcynomolgi_BAEJ01000249                        : AGGTGGCCTTTAGTAACGTGTTGCTCAAATCCTTCATCAATAAAAAAAACGAAGAAAATACATTTAATACGATCATACAG :  480
Brazil-I_AFMK01000508.1_A                      : AGGTGGCCTTTAGCAACGTCTTGCTCAAATCCTTCATCGATAAAAAAAATGAAGAAAACACATTTAACACGATCATACAG :  480
VCG-I                                          : AGGTGGCCTTTAGCAACGTCTTGCTCAAATCCTTCATCGATAAAAAAAATGAAGAAAACACATTTAACACGATCATACAG :  480
Sal-1_PVX_088910                               : AGGTGGCCTTTAGCAACGTCTTGCTCAAATCCTTCATCGATAAAAAAAATGAAGAAAACACATTTAACACGATCATACAG :  480
Mauritania_I_AFNI01000333                      : AGGTGGCCTTTAGCAACGTCTTGCTCAAATCCTTCATCGATAAAAAAAATGAAGAAAACACATTTAACACGATCATACAG :  480
NKorean_AFNJ01000531                           : AGGTGGCCTTTAGCAACGTCTTGCTCAAATCCTTCATCGATAAAAAAAATGAAGAAAACACATTTAACACGATCATACAG :  480
India-VII_AFBK01000586_AFBK01000587            : AGGTGGCCTTTAGCAACGTCTTGCTCAAATCCTTCATCGATAAAAAAAATGAAGAAAACACATTTAACACGATCATACAG :  480
                                                 AgGTGGCCTTtAgcAACGT TTGCTcAAATCcTTCATCgATAAaAA AA GAAGAAAAcACATTtAAcaCGATcATAcAG
```

```
                                                 *       500         *       520         *       540         *       560
P.coatneyi_CM002856.1                      : AATTACAAAGTTCTGTCCACTTGTATTGATGAAGATTTGAAGGATATTTACAATGCATCTATAGAATTATTCTCCGACAT :  560
Pknowlesi_NC_011906.1                      : AATTACAAAGTTTTGTCCACTTGTATTGATGAAGATTTGAAGGACATTTATAATGCATCCATAGAGTTATTTTCCGATCT :  560
Pinui_NW_008481888.1                       : AATTACAAAGTTCTGTCCACTTGTATTGATGCAGATTTGAAGGACATTTATAGTGCATCCATACAGTTATTCTCCGACCT :  560
Pfragile_NW_012192586.1                    : AATTACAAAATTCTGTCCACTTGTATTGATGAAGATTTGAAGGACATTTATGATGCGTCCATACAGTTATTCTCCGACAT :  560
Pcynomolgi_BAEJ01000249                    : AATTACAAACTTCTGTCCACTTGTATTGATGAAGATTTGAAGGACATTTATAATGCATCCATAGATTTATTCTCCGATAT :  560
Brazil-I_AFMK01000508.1_A                  : AATTACAAAGTTCTGTCCACTTGCATTGACGATGATTTGAAGGACATTTATAATGCATCCATAGAGTTATTCTCCGACAT :  560
VCG-I                                      : AATTACAAAGTTCTGTCCACTTGCATTGACGATGATTTGAAGGACATTTATAATGCATCCATAGAGTTATTCTCCGACAT :  560
Sal-1_PVX_088910                           : AATTACAAAGTTCTGTCCACTTGCATTGACGATGATTTGAAGGACATTTATAATGCATCCATAGAGTTATTCTCCGACAT :  560
Mauritania_I_AFNI01000333                  : AATTACAAAGTTCTGTCCACTTGCATTGACGATGATTTGAAGGACATTTATAATGCATCCATAGAGTTATTCTCCGACAT :  560
NKorean_AFNJ01000531                       : AATTACAAAGTTCTGTCCACTTGCATTGACGATGATTTGAAGGACATTTATAATGCATCCATAGAGTTATTCTCCGACAT :  560
India-VII_AFBK01000586_AFBK01000587        : AATTACAAAGTTCTGTCCACTTGCATTGACGATGATTTGAAGGACATTTATAATGCATCCATAGAGTTATTCTCCGACAT :  560
                                             AATTACAAAgTTcTGTCCACTTG ATTGA Ga GATTTGAAGGAcATTTAtaaTGCaTCcATAgAgTTATTcTCCGAcaT

                                                 *       580         *       600         *       620         *       640
P.coatneyi_CM002856.1                      : TAGAAGCTCCGTCACAGAAATCACGGAAAAGTTGTGGTCCAAAAATATGATCGATGTTTTAAAAACAAGAGAGCAAGCCA :  640
Pknowlesi_NC_011906.1                      : TAGAACATCTGTCAGAGAAATTACGGAAAAATTGTGGTCCAAAAATATGATCGAGGTTTTAAAAACAAGAGAGCAAGCGA :  640
Pinui_NW_008481888.1                       : TAGAAGCGCCGTCACAGAAATCTCGGAAAGGTTGTGGTCCAAGAATATGATCGATGTTTTAAAGACAAGAGAGGAAACCA :  640
Pfragile_NW_012192586.1                    : TAGAGTGTCCGTCACAGAAATCACAGAAAAGCTGTGGTCTAAAAATATGATCGACGTTTTAAAGACAAGAGAGCAAACCA :  640
Pcynomolgi_BAEJ01000249                    : TAGAACCTCCGTCACGGAAATCACGGAAAAGTTGTGGTCCAAAAATATGATCGAGGTTTTAAAGGCAAGAGAGCAAACCA :  640
Brazil-I_AFMK01000508.1_A                  : AAGAACCTCCGTCACAGAAATTACCGAAAAGTTGTGGTCCAAAAATATGATCGAAGTTTTAAAGACAAGAGAGCAAACCA :  640
VCG-I                                      : AAGAACCTCCGTCACAGAAATTACCGAAAAGTTGTGGTCCAAAAATATGATCGAAGTTTTAAAGACAAGAGAGCAAACCA :  640
Sal-1_PVX_088910                           : AAGAACCTCCGTCACAGAAATTACCGAAAAGTTGTGGTCCAAAAATATGATCGAAGTTTTAAAGACAAGAGAGCAAACCA :  640
Mauritania_I_AFNI01000333                  : AAGAACCTCCGTCACAGAAATTACCGAAAAGTTGTGGTCCAAAAATATGATCGAAGTTTTAAAGACAAGAGAGCAAACCA :  640
NKorean_AFNJ01000531                       : AAGAACCTCCGTCACAGAAATTACCGAAAAGTTGTGGTCCAAAAATATGATCGAAGTTTTAAAGACAAGAGAGCAAACCA :  640
India-VII_AFBK01000586_AFBK01000587        : AAGAACCTCCGTCACAGAAATTACCGAAAAGTTGTGGTCCAAAAATATGATCGAAGTTTTAAAGACAAGAGAGCAAACCA :  640
                                              AGAa ctCcGTCAcaGAAAT aC GAAAagtTGTGGTCcAAaAATATGATCGA GTTTTAAAgaCAAGAGAGcAAaCcA

                                                 *       660         *       680         *       700         *       720
P.coatneyi_CM002856.1                      : TTGCAGGCATTTTATGTGAGTTAAGAAACGGAAACAATTCTCCCCTAGTATCAAACAGTTTGTCGTATGAAAATTTTGGA :  720
Pknowlesi_NC_011906.1                      : TTGCAGGTATTTTATGTGAGTTAAGAAACGGAAATAATTCTACCCTAGTATCAAATAGTTTGTCGTATGAAAATTTTGGA :  720
Pinui_NW_008481888.1                       : TTACAGGCATTTTATGTGAGTTAAGAAAGGGAAATAATTCTACTCTAGTATCGAACAGTTTTTCGTATGACAATTTTGGA :  720
Pfragile_NW_012192586.1                    : TTGCAGGTATTTTATGTGAGTTACGAAATGGAAATAAATCAACCCTAGTATCAAACAGTTTGTCATATGAAAATTTTGGG :  720
Pcynomolgi_BAEJ01000249                    : TTGCAGGCATTTTATGTGAGTTAAGAAACGGAAATAATTCTACCCTAGTATCCAACAGTTTGTCGTATGAAAATTTTGGA :  720
Brazil-I_AFMK01000508.1_A                  : TTGCAGGCATTTTATGTGAGTTAAGAAATGGAAATAATTCTCCCCTAGTATCGAACAGTTTTTCCTATGAAAATTTTGGA :  720
VCG-I                                      : TTGCAGGCATTTTATGTGAGTTAAGAAATGGAAATAATTCTCCCCTAGTATCGAACAGTTTTTCCTATGAAAATTTTGGA :  720
Sal-1_PVX_088910                           : TTGCAGGCATTTTATGTGAGTTAAGAAATGGAAATAATTCTCCCCTAGTATCGAACAGTTTTTCCTATGAAAATTTTGGA :  720
Mauritania_I_AFNI01000333                  : TTGCAGGCATTTTATGTGAGTTAAGAAATGGAAATAATTCTCCCCTAGTATCGAACAGTTTTTCCTATGAAAATTTTGGA :  720
NKorean_AFNJ01000531                       : TTGCAGGCATTTTATGTGAGTTAAGAAATGGAAATAATTCTCCCCTAGTATCGAACAGTTTTTCCTATGAAAATTTTGGA :  720
India-VII_AFBK01000586_AFBK01000587        : TTGCAGGCATTTTATGTGAGTTAAGAAATGGAAATAATTCTCCCCTAGTATCGAACAGTTTTTCCTATGAAAATTTTGGA :  720
                                             TTgCAGGcATTTTATGTGAGTTAaGAAA GGAAAtAAtTCt CcCTAGTATC AAcAGTTT TC TATGAaAATTTTGGa
```

```
                                                     *      740         *      760         *      780         *      800
P.coatneyi_CM002856.1                         : ATCCTCAAGGTTAATTATGAAGCCTTAATGAACCAGGCGTATAAAGCTTTTTCAGACTACTATTCATACTTTCCCGCTTT :  800
Pknowlesi_NC_011906.1                         : ATCCTTAAAGTGAATTATGAAGGCTTAATAAACCAGGCGTATAAAGCCTTTTCAGACTACTATTCTTACTTTCCCGCGTT :  800
Pinui_NW_008481888.1                          : ATCCTCAAGGTTAATTATGAAGGGTTACTAAACCAGGCGTATGTGGCATTTTCAGACTACTATTCATTCTTTCCCCTTTT :  800
Pfragile_NW_012192586.1                       : ATCCTCAAGGTTAATTATGAAGGGTTAATGAACCAGGCGTACAAGGCATTTTCAGACTACTATTCATACTTTCCCGATTT :  800
Pcynomolgi_BAEJ01000249                       : ATCCTCAAGGTTAATTATGAAGAGTTACTAAACCAGGCGTATGCGGCGTTTTCAGACTACTATTCATATTTCCCCGCTTT :  800
Brazil-I_AFMK01000508.1_A                     : ATTCTCAAGGTTAATTATGAGGGATTACTAAACCAGGCGTATGCGGCCTTTTCAGACTACTATTCATACTTTCCCGCTTT :  800
VCG-I                                         : ATTCTCAAGGTTAATTATGAGGGATTACTAAACCAGGCGTATGCGGCCTTTTCAGACTACTATTCATACTTTCCCGCTTT :  800
Sal-1_PVX_088910                              : ATTCTCAAGGTTAATTATGAGGGATTACTAAACCAGGCGTATGCGGCCTTTTCAGACTACTATTCATACTTTCCCGCTTT :  800
Mauritania_I_AFNI01000333                     : ATTCTCAAGGTTAATTATGAGGGATTACTAAACCAGGCGTATGCGGCCTTTTCAGACTACTATTCATACTTTCCCGCTTT :  800
NKorean_AFNJ01000531                          : ATTCTCAAGGTTAATTATGAGGGATTACTAAACCAGGCGTATGCGGCCTTTTCAGACTACTATTCATACTTTCCCGCTTT :  800
India-VII_AFBK01000586_AFBK01000587           : ATTCTCAAGGTTAATTATGAGGGATTACTAAACCAGGCGTATGCGGCCTTTTCAGACTACTATTCATACTTTCCCGCTTT :  800
                                                AT CTCAAGGTtAATTATGA Gg TTA TaAACCAGGCGTAt  gGC TTTTCAGACTACTATTCaTacTTtCCCgctTT

                                                     *      820         *      840         *      860         *      880
P.coatneyi_CM002856.1                         : TGCCATTAAATTGTTAGAAAAGGGAGGGTTCGTCGAGCGATTGGTCGCTATCCATGAAAGCTTGACCAACTACAGGACGA :  880
Pknowlesi_NC_011906.1                         : TGCCATTAGACTTTTAGAAAAGGATGGGTTGGTCGAGCGGTTGGTCGCTATCCATGAAAGCTTGACCAACTACAGGACGA :  880
Pinui_NW_008481888.1                          : TGCCATGCGCTTGTTAGAAAATGGCGGATTGGTCGAGCGATTGGTCGCTATCCATGAGAAATTGACCAACTACAGGACGA :  880
Pfragile_NW_012192586.1                       : TGCCATTAAATTGTTGGAGAAAGGTGGGTTGGTCGAGCGGTTGGTCGCTATCCATGAGAGCTTGACCAACTACAGGACGA :  880
Pcynomolgi_BAEJ01000249                       : TGCCATTAAATTGTTAGAAAAGGGCGGGTTGGTCGAGCGGTTGGTCGCTATCCATGAGAGCTTAACCAACTACAGGACCA :  880
Brazil-I_AFMK01000508.1_A                     : TGCCATTAGCATGTTAGAAAAGGGAGGGTTGGTCGACCGCTTGGTCGCCATCCATGAGAGCTTGACCAACTACAGGACGA :  880
VCG-I                                         : TGCCATTAGCATGTTAGAAAAGGGAGGGTTGGTCGACCGCTTGGTCGCCATCCATGAGAGCTTGACCAACTACAGGACGA :  880
Sal-1_PVX_088910                              : TGCCATTAGCATGTTAGAAAAGGGAGGGTTGGTCGACCGCTTGGTCGCCATCCATGAGAGCTTGACCAACTACAGGACGA :  880
Mauritania_I_AFNI01000333                     : TGCCATTAGCATGTTAGAAAAGGGAGGGTTGGTCGACCGCTTGGTCGCCATCCATGAGAGCTTGACCAACTACAGGACGA :  880
NKorean_AFNJ01000531                          : TGCCATTAGCATGTTAGAAAAGGGAGGGTTGGTCGACCGCTTGGTCGCCATCCATGAGAGCTTGACCAACTACAGGACGA :  880
India-VII_AFBK01000586_AFBK01000587           : TGCCATTAGCATGTTAGAAAAGGGAGGGTTGGTCGACCGCTTGGTCGCCATCCATGAGAGCTTGACCAACTACAGGACGA :  880
                                                TGCCATta   TgTTaGAaAAgGg GGgTTgGTCGA CG TTGGTCGC ATCCATGAgAgcTTgACCAACTACAGGACgA

                                                     *      900         *      920         *      940         *      960
P.coatneyi_CM002856.1                         : AAAATATTCTCAAGAAGATCAATGAGAAGTCCAAAAATGAGGTCCTGAATAACGAGGATATTATGCACAGCTTGAGCAGT :  960
Pknowlesi_NC_011906.1                         : GAAATATTCTCAAGAAGATCAATGAGAAGTCCAAAAATGAGGTCCTGAATAATGAGGATATTATGCACAGCTTGAGCAGT :  960
Pinui_NW_008481888.1                          : GAAATATTCTCAAGAAGATCAATGACAAGTCCAAAAATGAGGTCCTGAATAACGAGGATATTATGCACAGCTTGAGCAGT :  960
Pfragile_NW_012192586.1                       : AAAATATTCTCCAGAAAATCAATGAGAAGTCAAAAAATGAAGTCCTGAATAACGAGGATATTATGCACAGCTTGAGCAGT :  960
Pcynomolgi_BAEJ01000249                       : GAAATATTCTCAAGAAGATCAATGAAAAGTCCAAAAATGAGGTCCTGAATAATGGGGATATTATGCACAGCTTGAGCAGT :  960
Brazil-I_AFMK01000508.1_A                     : GAAATATTCTCAAGAAGATCAATGAGAAGTCCAAAAATGAGGTCCTCAATAATGAAGAAATTATGCACAGCTTGAGCAGT :  960
VCG-I                                         : GAAATATTCTCAAGAAGATCAATGAGAAGTCCAAAAATGAGGTCCTCAATAATGAAGAAATTATGCACAGCTTGAGCAGT :  960
Sal-1_PVX_088910                              : GAAATATTCTCAAGAAGATCAATGAGAAGTCCAAAAATGAGGTCCTCAATAATGAAGAAATTATGCACAGCTTGAGCAGT :  960
Mauritania_I_AFNI01000333                     : GAAATATTCTCAAGAAGATCAATGAGAAGTCCAAAAATGAGGTCCTCAATAATGAAGAAATTATGCACAGCTTGAGCAGT :  960
NKorean_AFNJ01000531                          : GAAATATTCTCAAGAAGATCAATGAGAAGTCCAAAAATGAGGTCCTCAATAATGAAGAAATTATGCACAGCTTGAGCAGT :  960
India-VII_AFBK01000586_AFBK01000587           : GAAATATTCTCAAGAAGATCAATGAGAAGTCCAAAAATGAGGTCCTCAATAATGAAGAAATTATGCACAGCTTGAGCAGT :  960
                                                gAAATATTCTCaAGAAgATCAATGAgAAGTCcAAAAATGAgGTCCT AATAA Ga GA ATTATGCACAGCTTGAGCAGT
```

```
                                                       *       980         *      1000         *      1020         *      1040
P.coatneyi_CM002856.1                      : TACAAGCACCATGCCGGGGGCACACGTGGCTCCTTCGTGCAGTCCAGAGGTGTGAGCCAAGTTACGCAAGGCGTCCTGAG : 1040
Pknowlesi_NC_011906.1                      : TATAAGCACCACGCCGGGGGTACTCGTGGCTCCTTCATGCAGTACAGAGGTGTGGGCCAACTTTCGAAGGCGGCCTCAG : 1040
Pinui_NW_008481888.1                       : TACAAGCACCATGCCACGGGTACGCGTGGCTCCTTCCTGCAGTCCAGAGGTGTGCGCCAAGTTACGAACAGCAACGTGAG : 1040
Pfragile_NW_012192586.1                    : TACAAGCACCGTGCCGGGAGTACGCGTGGTTCCTTCCTGCAGTCCAGAGGTGCGCACAATGTTACAAAAGTCGATGTGAA : 1040
Pcynomolgi_BAEJ01000249                    : TACAAGCACCATGCTGGGGGCACGCGTGGCTCCTTCCTGCAGTCCAGAGTTGCGCGCGAAGTTAGGCAAGGCGACGTGAG : 1040
Brazil-I_AFMK01000508.1_A                  : TACAAGCACCATGCCGGGGGCACGCGTGGCGCCTTCCTGCAGTCCAGAGATGTGCGCGAAGTTACGCAAGGAGATGTGAG : 1040
VCG-I                                      : TACAAGCACCATGCCGGGGGCACGCGTGGCGCCTTCCTGCAGTCCAGAGATGTGCGCGAAGTTACGCAAGGAGATGTGAG : 1040
Sal-1_PVX_088910                           : TACAAGCACCATGCCGGGGGCACGCGTGGCGCCTTCCTGCAGTCCAGAGATGTGCGCGAAGTTACGCAAGGAGATGTGAG : 1040
Mauritania_I_AFNI01000333                  : TACAAGCACCATGCCGGGGGCACGCGTGGCGCCTTCCTGCAGTCCAGAGATGTGCGCGAAGTTACGCAAGGAGATGTGAG : 1040
NKorean_AFNJ01000531                       : TACAAGCACCATGCCGGGGGCACGCGTGGCGCCTTCCTGCAGTCCAGAGATGTGCGCGAAGTTACGCAAGGAGATGTGAG : 1040
India-VII_AFBK01000586_AFBK01000587        : TACAAGCACCATGCCGGGGGCACGCGTGGCGCCTTCCTGCAGTCCAGAGATGTGCGCGAAGTTACGCAAGGAGATGTGAG : 1040
                                             TAcAAGCACCatGCcggGgG ACgCGTGGc CCTTCcTGCAGTcCAGAG TGtGcgC AagTTacg Aagg ga gTgAg

                                                       *      1060         *      1080         *      1100         *      1120
P.coatneyi_CM002856.1                      : CGTCGACGAGAAGGGTGACCCGTCCTCCACCGCGGGG---GGCAACCAAAGCGCAAACATGGCCACGGCGGCCCCCAAC- : 1116
Pknowlesi_NC_011906.1                      : CGTTGACGAGAAGGGTGGCCAGCAGATTGCCTCCGCGGTGGGCAACCAAAGCGCAAACATGGTCGCGCCGGCTCCTAAGG : 1120
Pinui_NW_008481888.1                       : CGTTGACCAGAAGGGCGATCAGACCGCCACCGCGGGG---GGCAACGAAAGCGAAAACGTGCCCGCGGCGGTCCCCAAGG : 1117
Pfragile_NW_012192586.1                    : GGTTGACGAG------------ACCTCCACCACGGGG---GGCAACCAAAGCGCAAACATGGCCGCTGCGGCGCCTAACA : 1105
Pcynomolgi_BAEJ01000249                    : CGTTGACGAGAAGGGTGACCAGACTGCCACCACAGGG---GGCAACCAAAGCGCAAACATGGCCGCGGCGGCCCCCAAGG : 1117
Brazil-I_AFMK01000508.1_A                  : CGTTGATGAGAAGGGCGACCGGGCCACCACCGCGGGG---GGCAACCAAAGCGCAAGCGTGGCTGCGGCGGCCCCGAAGG : 1117
VCG-I                                      : CGTTGATGAGAAGGGCGACCGGGCCACCACCGCGGGG---GGCAACCAAAGCGCAAGCGTGGCTGCGGCGGCCCCGAAGG : 1117
Sal-1_PVX_088910                           : CGTTGATGAGAAGGGCGACCGGGCCACCACCGCGGGG---GGCAACCAAAGCGCAAGCGTGGCTGCGGCGGCCCCGAAGG : 1117
Mauritania_I_AFNI01000333                  : CGTTGATGAGAAGGGCGACCGGGCCACCACCGCGGGG---GGCAACCAAAGCGCAAGCGTGGCTGCGGCGGCCCCGAAGG : 1117
NKorean_AFNJ01000531                       : CGTTGATGAGAAGGGCGACCGGGCCACCACCGCGGGG---GGCAACCAAAGCGCAAGCGTGGCTGCGGCGGCCCCGAAGG : 1117
India-VII_AFBK01000586_AFBK01000587        : CGTTGATGAGAAGGGCGACCGGGCCACCACCGCGGGG---GGCAACCAAAGCGCAAGCGTGGCTGCGGCGGCCCCGAAGG : 1117
                                             cGTtGA gAGaaggg gacc g cc ccaCC CgGgG   GGCAACcAAAGCGcAA C TGgc gCggCGGccCC AAgg

                                                       *      1140         *      1160         *      1180         *      1200
P.coatneyi_CM002856.1                      : --------------------------------------ACGGTTCCTGCTACCAACACGGCTGCTGCTACCACTGCTACT : 1158
Pknowlesi_NC_011906.1                      : ATTCCTCCCCTACAATG---------------------GCTGCTCCTTCCACCGACACTGTTACTAACAACATGGCTACC : 1179
Pinui_NW_008481888.1                       : ATGACGCGCCTACTAGGGTTGCTTCTCCCGGCCCTGCTGCTTTTCCCAACACGGTTGCTTCCAACCCTACTGCTGTTTCC : 1197
Pfragile_NW_012192586.1                    : ATTCGGGCCCTACCACCGCTGCTGCTGCAAACAGTGCTGCTTCCCCAAACACGGCAGCAACTACCAACACTGCGGCAACT : 1185
Pcynomolgi_BAEJ01000249                    : ATGCGAGCCCTACCACG---GCTGCTTCCAACGCTGCTGCTTCCCCCGACACTACTGCTGCGAACACTGCTGCTGCGAAC : 1194
Brazil-I_AFMK01000508.1_A                  : ATGCGGGCCCAACCGTG---------------------GCTGCTCCTAACACTGCTGCTACGCTCAAAACGGCT------ : 1170
VCG-I                                      : ATGCGGGCCCAACCGTG---------------------GCTGCTCCTAACACTGCTGCTACGCTCAAAACGGCT------ : 1170
Sal-1_PVX_088910                           : ATGCGGGCCCAACCGTG---------------------GCTGCTCCTAACACTGCTGCTACGCTCAAAACGGCT------ : 1170
Mauritania_I_AFNI01000333                  : ATGCGGGCCCAACCGTG---------------------GCTGCTCCTAACACTGGTGCTACGCTCAAAACGGCT------ : 1170
NKorean_AFNJ01000531                       : ATGCGGGCCCAACCGTG---------------------GCTGCTCCTAACACTGCTGCTACGCTCAAAACGGCT------ : 1170
India-VII_AFBK01000586_AFBK01000587        : ATGCGGGCCCAACCGTG---------------------GCTGCTCCTAACACTGCTGCTACGCTCAAAACGGCT------ : 1170
                                             at c    ccc ac    g                     gCt ctCC   acAC g  gCt c    ca   ac gct
```

```
                                               *      1220         *      1240         *      1260         *      1280
P.coatneyi_CM002856.1               : ACTACGAACACGGCAACTAATACCAACACGGCA------------------ACTAATACCAACACGGCTACT---AATAC : 1217
Pknowlesi_NC_011906.1               : ACTACAACCGCGCCTGCTACTGCCAACATGGCTGCTCCTTCCACCAACACTGCTACTGCCAACATGGCTGCT---CCTTC : 1256
Pinui_NW_008481888.1                : AACGCTACTGCTGCTTCCAACGCTACTGCTGCTTCCAACGCTACTGCTGCTTCCAACGCTACTGAT---------GCTTC : 1268
Pfragile_NW_012192586.1             : ACCAGC---------------------------------------------------------------------GCTTC : 1196
Pcynomolgi_BAEJ01000249             : ACCGCTGCTGCGAACACCGCTGCTGCTCCGAACACTGCTTCTGCTTCCAACGCTGCTGCTACTCCCAACCCTGCTGCTGC : 1274
Brazil-I_AFMK01000508.1_A           : ---------------------------------------------------------------------------GCTTC : 1175
VCG-I                               : ---------------------------------------------------------------------------GCTTC : 1175
Sal-1_PVX_088910                    : ---------------------------------------------------------------------------GCTTC : 1175
Mauritania_I_AFNI01000333           : ---------------------------------------------------------------------------GCTTC : 1175
NKorean_AFNJ01000531                : ---------------------------------------------------------------------------GCTTC : 1175
India-VII_AFBK01000586_AFBK01000587 : ---------------------------------------------------------------------------GCTTC : 1175
                                                                                                              gcTtC

                                               *      1300         *      1320         *      1340         *      1360
P.coatneyi_CM002856.1               : CAACAGTGCAGCTACTACCAACACGGCCGCT------------------ACTACCAACACGGCCGCTACTACCAACACGA : 1279
Pknowlesi_NC_011906.1               : CACCAACACTGCTACTGCCAACATGGCTGCTCCTTCCACCGACACTGTAACTACCAACATGGCTACCACTACAACCGTGC : 1336
Pinui_NW_008481888.1                : CAACGCTACTGCTGCTTCCAACCCTGCTGCT------------------------------------------------C : 1300
Pfragile_NW_012192586.1             : CAACGATACTAATGCTTCTCCCCACACGAAT------------------------------------------------A : 1228
Pcynomolgi_BAEJ01000249             : TCCGAACACTGCTGCTACTCCCAACTCTGCT------------------------------------------------A : 1306
Brazil-I_AFMK01000508.1_A           : CCCCAACGCGGCTGCTACTAACACTGCTGCT------------------------------------------------C : 1207
VCG-I                               : CCCCAACGCGGCTGCTACTAACACTGCTGCT------------------------------------------------C : 1207
Sal-1_PVX_088910                    : CCCCAACGCGGCTGCTACTAACACTGCTGCT------------------------------------------------C : 1207
Mauritania_I_AFNI01000333           : CCCCAACGCGGCTGCTACTAACACTGCTGCT------------------------------------------------C : 1207
NKorean_AFNJ01000531                : CCCCAACGCGGCTGCTACTAACACTGCTGCT------------------------------------------------C : 1207
India-VII_AFBK01000586_AFBK01000587 : CCCCAACGCGGCTGCTACTAACACTGCTGCT------------------------------------------------C : 1207
                                      c  caa  C gcTgCT C aaCa  gCtgcT

                                               *      1380         *      1400         *      1420         *      1440
P.coatneyi_CM002856.1               : CCGCTACTACCAGTGTAGCTACTTCCCCCTGAACACC----------------------CCCCTGTACGGCACCAGCTCC : 1338
Pknowlesi_NC_011906.1               : CTGCTACTGCCAACACGGCTGCTTCCCCC---GACACTAATACTGATTCTTCTACTTACCCCCTGTATGGCACGGAATCC : 1413
Pinui_NW_008481888.1                : CTCCCAACACTGCTGCT---ACTTCCCAA---CTGAGCAAC------------------CCCCTGTATGGCATCAGCTCC : 1356
Pfragile_NW_012192586.1             : CTTCCAACGAGGGTGCTTCCACCAGTCCC---CTGAGCAAC------------------CCCCTGTACGGCACCCGCTAC : 1287
Pcynomolgi_BAEJ01000249             : CCCCCAACGCTGCTGCT---ACTTCCCCA---CTGAGCACC------------------CCCCTGTACGACACAAGCTCC : 1362
Brazil-I_AFMK01000508.1_A           : CCCCCAACATGGGTGCC---ACCTCCCCG---CTGAGCAAC------------------CCCCTGTACGGCACCAGCTCC : 1263
VCG-I                               : CCCCCAACATGGGTGCC---ACCTCCCCG---CTGAGCAAC------------------CCCCTGTACGGCACCAGCTCC : 1263
Sal-1_PVX_088910                    : CCCCCAACATGGGTGCC---ACCTCCCCG---CTGAGCAAC------------------CCCCTGTACGGCACCAGCTCC : 1263
Mauritania_I_AFNI01000333           : CCCCCAACATGGGTGCC---ACCTCCCCG---CTGAGCAAC------------------CCCCTGTACGGCACCAGCTCC : 1263
NKorean_AFNJ01000531                : CCCCCAACATGGGTGCC---ACCTCCCCG---CTGAGCAAC------------------CCCCTGTACGGCACCAGCTCC : 1263
India-VII_AFBK01000586_AFBK01000587 : CCCCCAACATGGGTGCC---ACCTCCCCG---CTGAGCAAC------------------CCCCTGTACGGCACCAGCTCC : 1263
                                      C  CcAac   g tgc    aC tccCc    ctgAgcaac                  CCCCTGTAcGgCAccagcTcC
```

```
                                                        *      1460       *      1480       *      1500       *      1520
P.coatneyi_CM002856.1                         : TCGAGGACAAAGGACGTCGTAGTGCTGGTCAGAGATCTGCTCAAAAACACAAACATCATAAAATTCGAGAAGAACGAACC : 1418
Pknowlesi_NC_011906.1                         : TCGAAGACAAAGGACGTCGTAGTTCTAGTCAGAGATCTTCTCAAAGACACAAACATCATCAAGTTCGAGAAGAACGAACC : 1493
Pinui_NW_008481888.1                          : TTGAATACAAAGGACGTCGTAGTGCTGGTCAGAGATCTGCTCAAGAACACAAACATCATCAAGTTCGACAAGGACGAACC : 1436
Pfragile_NW_012192586.1                       : TTGAAGACAAAGGACGTCGTAGTGCTCATCAGAGATCTGCTCAAAAACACAAACATCATCAAGTTCGAGAAGGACGAACC : 1367
Pcynomolgi_BAEJ01000249                       : TTGAAGACAAAGGACGTCGTAGTGCTCGTCAGAGATCTGCTCAAAAACACAAACATCATCAAGTTCGAGAAGGACGAACC : 1442
Brazil-I_AFMK01000508.1_A                     : CTGCAGCCAAAGGACGTCGCGGTGCTGGTCAGAGATCTGCTCAAGAACACGAACATCATCAAGTTCGAGAATAACGAACC : 1343
VCG-I                                         : CTGCAGCCAAAGGACGTCGCGGTGCTGGTCAGAGATCTGCTCAAGAACACGAACATCATCAAGTTCGAGAATAACGAACC : 1343
Sal-1_PVX_088910                              : CTGCAGCCAAAGGACGTCGCGGTGCTGGTCAGAGATCTGCTCAAGAACACGAACATCATCAAGTTCGAGAATAACGAACC : 1343
Mauritania_I_AFNI01000333                     : CTGCAGCCAAAGGACGTCGCGGTGCTGGTCAGAGATCTGCTCAAGAACACGAACATCATCAAGTTCGAGAATAACGAACC : 1343
NKorean_AFNJ01000531                          : CTGCAGCCAAAGGACGTCGCGGTGCTGGTCAGAGATCTGCTCAAGAACACGAACATCATCAAGTTCGAGAATAACGAACC : 1343
India-VII_AFBK01000586_AFBK01000587           : CTGCAGCCAAAGGACGTCGCGGTGCTGGTCAGAGATCTGCTCAAGAACACGAACATCATCAAGTTCGAGAATAACGAACC : 1343
                                                 tG ag CAAAGGACGTCG GTgCT gTCAGAGATCTgCTCAA aACAC AACATCATcAAgTTCGAgAA ACGAACC

                                                        *      1540       *      1560       *      1580       *      1600
P.coatneyi_CM002856.1                         : GACTAGCCAAATAGACGATGAAGGAATTAAGAAGCTCATAGAGAGTTCCTTCTTCGACCTAAGCGACAACACCATGTTAA : 1498
Pknowlesi_NC_011906.1                         : GACTAGCCAAATAGACGATGAAGGAATTAAGAAGCTCATAGAGAGTTCGTTCTTCGACTTGAGCGACAACACCATGTTAA : 1573
Pinui_NW_008481888.1                          : GACTAGTCAAATGGACGATGAAGGAATTAAGAAGCTCATAGAGAGTTCCTTCTTCGACTTGAGCGACAACACCATGTTAA : 1516
Pfragile_NW_012192586.1                       : AACTGGCCAAATTGACCATGAAGGAATTAAAAAACTCATAGAAAGTTCCTTCTTCGACCTGAGCGACAACACCATGTTAA : 1447
Pcynomolgi_BAEJ01000249                       : GACTAGCCAAATAGACGAGGAAGGAATTAAGAAGCTCATAGAGAGTTCCTTCTTCGACTTGAGCGACAACACCATGTTAA : 1522
Brazil-I_AFMK01000508.1_A                     : GACTAGCCAAATGGACGATGAAGAAATTAAGAAGCTCATTGAGAGCTCCTTTTTCGACTTGAGCGACAACACCATGTTAA : 1423
VCG-I                                         : GACTAGCCAAATGGACGATGAAGAAATTAAGAAGCTCATTGAGAGCTCCTTTTTCGACTTGAGCGACAACACCATGTTAA : 1423
Sal-1_PVX_088910                              : GACTAGCCAAATGGACGATGAAGAAATTAAGAAGCTCATTGAGAGCTCCTTTTTCGACTTGAGCGACAACACCATGTTAA : 1423
Mauritania_I_AFNI01000333                     : GACTAGCCAAATGGACGATGAAGAAATTAAGAAGCTCATTGAGAGCTCCTTTTTCGACTTGAGCGACAACACCATGTTAA : 1423
NKorean_AFNJ01000531                          : GACTAGCCAAATGGACGATGAAGAAATTAAGAAGCTCATTGAGAGCTCCTTTTTCGACTTGAGCGACAACACCATGTTAA : 1423
India-VII_AFBK01000586_AFBK01000587           : GACTAGCCAAATGGACGATGAAGAAATTAAGAAGCTCATTGAGAGCTCCTTTTTCGACTTGAGCGACAACACCATGTTAA : 1423
                                                gACTaGcCAAAT GACgAtGAAG AATTAAgAAgCTCAT GAgAG TCcTT TTCGACtTgAGCGACAACACCATGTTAA

                                                        *      1620       *      1640       *      1660       *      1680
P.coatneyi_CM002856.1                         : TGCGCTTGCTCATAAAGCCGCAGGCAGCCATCCTATTCATCATTCAGTCCTTCATTATGATGACGCCGTCCCCCACAAGA : 1578
Pknowlesi_NC_011906.1                         : TGCGATTGATCATAAAGCCGCAGGCTTCCATCTTATTCATTATCCAGTCCTTCATTATGATGACGCCATCCCCCACGAGA : 1653
Pinui_NW_008481888.1                          : TGCAGTTGCTCATAAAGCCGCAGGCAGCCATGTTATTCATCATTCAGTCCTTCATTATGATGACACCCTCCCCCACGCGA : 1596
Pfragile_NW_012192586.1                       : TGAGGTTGCTCATAAAGCCGCAGGCAGCCATCTTATTCATCATTCAGTCCTTTATCATGATGACACCGTCGCCCACAAGA : 1527
Pcynomolgi_BAEJ01000249                       : TGCGATTGCTCATAAAGCCGCAGGCAGCCATCTTATTCATCATTCAGTCCTTCATTATGATGACGCCGTCCCCCACGCGA : 1602
Brazil-I_AFMK01000508.1_A                     : TGCGGTTGCTCATAAAGCCGCAGGCGGCCATCTTACTAATCATTGAGTCCTTCATTATGATGACGCCCTCCCCCACGAGG : 1503
VCG-I                                         : TGCGGTTGCTCATAAAGCCGCAGGCGGCCATCTTACTAATCATTGAGTCCTTCATTATGATGACGCCCTCCCCCACGAGG : 1503
Sal-1_PVX_088910                              : TGCGGTTGCTCATAAAGCCGCAGGCGGCCATCTTACTAATCATTGAGTCCTTCATTATGATGACGCCCTCCCCCACGAGG : 1503
Mauritania_I_AFNI01000333                     : TGCGGTTGCTCATAAAGCCGCAGGCGGCCATCTTACTAATCATTGAGTCCTTCATTATGATGACGCCCTCCCCCACGAGG : 1503
NKorean_AFNJ01000531                          : TGCGGTTGCTCATAAAGCCGCAGGCGGCCATCTTACTAATCATTGAGTCCTTCATTATGATGACGCCCTCCCCCACGAGG : 1503
India-VII_AFBK01000586_AFBK01000587           : TGCGGTTGCTCATAAAGCCGCAGGCGGCCATCTTACTAATCATTGAGTCCTTCATTATGATGACGCCCTCCCCCACGAGG : 1503
                                                TGcg TTGcTCATAAAGCCGCAGGC gCCATctTA T ATcATt AGTCCTTcATtATGATGACgCC TCcCCCACgaG
```

```
                                                      *       1700         *       1720         *       1740         *       1760
P.coatneyi_CM002856.1                         : GACGCCAAAATGTATTGCAAGAAAAAGCTAGTTAATGGCCAGCTAATAGAAAACTCCGACTTAAAAGCTGCGACGGAGGA : 1658
Pknowlesi_NC_011906.1                         : GACGCCAGAATGTATTGCAAGAAAAAACTAGTTAATGGCCAGTTAATAGAAAACAACGATTTAAAGGCTGAGACAGAGGA : 1733
Pinui_NW_008481888.1                          : GACGCCAAAACCTATTGCAACAAAAGCCTAGTTAATGGCAAGCTAATCGAAACCTCCGATATAAAGGCTTCGACGGAGGA : 1676
Pfragile_NW_012192586.1                       : GACGCCAAAATTTATTGCAAGAAAAATCTAGTTAATGGCCAGCTAGTACAAACCTCTGATTTCAGCGATGCGCACGAGGA : 1607
Pcynomolgi_BAEJ01000249                       : GACGCCAAAATCTATTGCAAGAAAGTCCTAGTTAATGGTCAGCTAGTCGAAACCTCCGATTTAAGCGCTGCGACGGAGGA : 1682
Brazil-I_AFMK01000508.1_A                     : GACGCCAAGACCTATTGCAAGAAAGCCCTAGTTAATGGCCAGCTAATCGAAACCTCAGATTTAAACGCGGCGACGGAGGA : 1583
VCG-I                                         : GACGCCAAGACCTATTGCAAGAAAGCCCTAGTTAATGGCCAGCTAATCGAAACCTCAGATTTAAACGCGGCGACGGAGGA : 1583
Sal-1_PVX_088910                              : GACGCCAAGACCTATTGCAAGAAAGCCCTAGTTAATGGCCAGCTAATCGAAACCTCAGATTTAAACGCGGCGACGGAGGA : 1583
Mauritania_I_AFNI01000333                     : GACGCCAAGACCTATTGCAAGAAAGCCCTAGTTAATGGCCAGCTAATCGAAACCTCAGATTTAAACGCGACGACGGAGGA : 1583
NKorean_AFNJ01000531                          : GACGCCAAGACCTATTGCAAGAAAGCCCTAGTTAATGGCCAGCTAATCGAAACCTCAGATTTAAACGCGGCGACGGAGGA : 1583
India-VII_AFBK01000586_AFBK01000587           : GACGCCAAGACCTATTGCAAGAAAGCCCTAGTTAATGGCCAGCTAATCGAAACCTCAGATTTAAACGCGGCGACGGAGGA : 1583
                                                GACGCCAa A  TATTGCAAgAAA   CTAGTTAATGGccAGcTAaT gAAAcCtc GAttTaAa Gc gcGacgGAGGA

                                                      *       1780         *       1800         *       1820         *       1840
P.coatneyi_CM002856.1                         : AGAAGACATGATAACCGAGTTTTCCAGCAAGTACAATTTATTCTACGAAAGGCTGAAACTGGAGGAGTTGCGCGAAATTG : 1738
Pknowlesi_NC_011906.1                         : AGAAGATATGATAAACGAATTTTCCAGTAAGTACAATTTATTCTATGAAAGGCTTAAGATGGAGGAGTTGCGCGAAATTG : 1813
Pinui_NW_008481888.1                          : AGAAGACCTGATAACCGAGTTTTCCAGCAAGTACAATTTATTCTACGAAAAGCTGAAGATGGAAGAGTTGCGAGAAATTG : 1756
Pfragile_NW_012192586.1                       : AGAAGATCTGATAAACGAATTTTCCAGCAAATACAATTTATTCTACGAAAGGTTGAAGCTGGAGGAGTTGCGCGAAATTG : 1687
Pcynomolgi_BAEJ01000249                       : AGAAGATCTGATAAATGAGTTTTCCAGCAAGTACAATTTATTCTACGAAAGGCTGAAACTGGAGGAGTTGCGCGAAATTG : 1762
Brazil-I_AFMK01000508.1_A                     : AGACGACCTCATAAACGAGTTTTCCAGCAGGTACAATTTATTCTACGAGAGGCTCAAGCTGGAGGAGTTGCGCGAAATTG : 1663
VCG-I                                         : AGACGACCTCATAAACGAGTTTTCCAGCAGGTACAATTTATTCTACGAGAGGCTCAAGCTGGAGGAGTTGCGCGAAATTG : 1663
Sal-1_PVX_088910                              : AGACGACCTCATAAACGAGTTTTCCAGCAGGTACAATTTATTCTACGAGAGGCTCAAGCTGGAGGAGTTGCGCGAAATTG : 1663
Mauritania_I_AFNI01000333                     : AGACGACCTCATAAACGAGTTTTCCAGCAGGTACAATTTATTCTACGAGAGGCTCAAGCTGGAGGAGTTGCGCGAAATTG : 1663
NKorean_AFNJ01000531                          : AGACGACCTCATAAACGAGTTTTCCAGCAGGTACAATTTATTCTACGAGAGGCTCAAGCTGGAGGAGTTGCGCGAAATTG : 1663
India-VII_AFBK01000586_AFBK01000587           : AGACGACCTCATAAACGAGTTTTCCAGCAGGTACAATTTATTCTACGAGAGGCTCAAGCTGGAGGAGTTGCGCGAAATTG : 1663
                                                AGA GA cT ATAAacGAgTTTTCCAGcA gTACAATTTATTCTAcGA AgGcT AAgcTGGAgGAGTTGCGcGAAATTG

                                                      *       1860         *       1880         *       1900         *       1920
P.coatneyi_CM002856.1                         : AACAGAACAGGAAAGCGCTCAAGAATTCGAAGGGAAGTCTGTCCGTGCTGCAGGTCGGCAACTCGCAGAATGGTCCAGAC : 1818
Pknowlesi_NC_011906.1                         : AACAGGACAGAAAATCGCTAAAGAATTCCAAGGGCAACTTGTCTGTGTTGGAGGTGCGCAACTCCCAGAATGGTCCCGAC : 1893
Pinui_NW_008481888.1                          : AAGAGAACAAGAAAGCTATCAAGAATTCGAAGGGCAGCCTGTCCGTGCTGGAGGTCGCCAACTCGCAAAATAGTCCCGAC : 1836
Pfragile_NW_012192586.1                       : AACAGAACAGGAAAGCGCTCAAGAATGCGAAGGGTAGCCTCTCCGTGCTGCAGGTCAGCAATTCGCAGAATGGTACCCAC : 1767
Pcynomolgi_BAEJ01000249                       : AACAGAACAGGAAAGCGCTCAAGAATTCGAAGGGCAGCCTGTCCGTGCTGGAGGTAGGCAACTCGCAGAATGGTCCAGAC : 1842
Brazil-I_AFMK01000508.1_A                     : AACAGAACAGGAAAGCGCTCAAGAATTCGAAGGGCACCCTGTCCGTGCTGGAGGTCGCCAACTCGCAGAACGCCCCCGAC : 1743
VCG-I                                         : AACAGAACAGGAAAGCGCTCAAGAATTCGAAGGGCACCCTGTCCGTGCTGGAGGTCGCCAACTCGCAGAACGCCCCCGAC : 1743
Sal-1_PVX_088910                              : AACAGAACAGGAAAGCGCTCAAGAATTCGAAGGGCACCCTGTCCGTGCTGGAGGTCGCCAACTCGCAGAACGCCCCCGAC : 1743
Mauritania_I_AFNI01000333                     : AACAGAACAGGAAAGCGCTCAAGAATTCGAAGGGCACCCTGTCCGTGCTGGAGGTCGCCAACTCGCAGAACGCCCCCGAC : 1743
NKorean_AFNJ01000531                          : AACAGAACAGGAAAGCGCTCAAGAATTCGAAGGGCACCCTGTCCGTGCTGGAGGTCGCCAACTCGCAGAACGCCCCCGAC : 1743
India-VII_AFBK01000586_AFBK01000587           : AACAGAACAGGAAAGCGCTCAAGAATTCGAAGGGCACCCTGTCCGTGCTGGAGGTCGCCAACTCGCAGAACGCCCCCGAC : 1743
                                                AAcAGaACAggAAAgCgcTcAAGAATtCgAAGGGcA ccTgTCcGTGcTGgAGGTcg CAAcTCgCAgAA g  cCcgAC
```

```
                                                    *      1940         *      1960         *      1980         *      2000
P.coatneyi_CM002856.1                    : GGGAAGGAGGCCAACGGAAGTGGAAACGCCGCAAATGGA----------------------------------------- : 1857
Pknowlesi_NC_011906.1                    : GGGAAGGAAGTCAACGGAAGCGGAGATGCCGCAAATGGA----------------------------------------- : 1932
Pinui_NW_008481888.1                     : GGGAAGAGGGTCAACGGAAGCGGAAACGACGCGAATGGA----------------------------------------- : 1875
Pfragile_NW_012192586.1                  : GGGAACGGGGTCAATCAAGGTGGACACGCTGCAAATGAA----------------------------------------- : 1806
Pcynomolgi_BAEJ01000249                  : GGGAGCGGAAACAACGGAAGCGGAAACGCCGCAAATGGA----------------------------------------- : 1881
Brazil-I_AFMK01000508.1_A                : GGGAAGGGCGTCAACGGGAGCGGAAACGCAGCAAATGCGAACGCCGCAAACGCAAACGCCGCAAACGCAAACGCCGCTAA : 1823
VCG-I                                    : GGGAAGGGCGTCAACGGGAGCGGAAACGCAGCAAATGCGAACGCCGCAAACGCAAACGCCGCAAACGCAAACGCCGCTAA : 1823
Sal-1_PVX_088910                         : GGGAAGGGCGTCAACGGGAGCGGAAACGCAGCAAATGCGAACGCCGCAAACGCAAACGCCGCAAACGCAAACGCCGCTAA : 1823
Mauritania_I_AFNI01000333                : GGGAAGGGCGTCAACGGGAGCGGAAACGCAGCAAATGCC----------------------------------------- : 1782
NKorean_AFNJ01000531                     : GGGAAGGGCGTCAACGGGAGCGGAAACGCAGCAAATGCGAACGCCGCAAACGCAAACGCCGCAAACGCAAACGCCGCTAA : 1823
India-VII_AFBK01000586_AFBK01000587      : GGGAAGGGCGTCAACGGGAGCGGAAACGCAGCAAATGCGAACGCCGCAAACGCAAACGCCGCAAACGCAAACGCCGCTAA : 1823
                                           GGGAaggg gtCAAcgg aGcGGAaAcGc GCaAATG

                                                    *      2020         *      2040         *      2060         *      2080
P.coatneyi_CM002856.1                    : -------------------------------------------------------------------------------- :    -
Pknowlesi_NC_011906.1                    : -------------------------------------------------------------------------------- :    -
Pinui_NW_008481888.1                     : -------------------------------------------------------------------------------- :    -
Pfragile_NW_012192586.1                  : -------------------------------------------------------------------------------- :    -
Pcynomolgi_BAEJ01000249                  : -------------------------------------------------------------------------------- :    -
Brazil-I_AFMK01000508.1_A                : TGCAAACGCCGCAAATGGGAACCTCGCAAATGCAAACCTCGCAAACGCAAAC---------------------------- : 1875
VCG-I                                    : TGCAAACGCCGCAAATGGGAACCTCGCAAATGCAAACCTCGCAAACGCAAACGCCGCAAACGCAGACGCCGCAAACGCAA : 1903
Sal-1_PVX_088910                         : TGCAAACGCCGCAAATGGGAACCTCGCAAATGCAAACCTCGCAAACGCAAACGCCGCAAACGCAGACGCCGCAAACGCAA : 1903
Mauritania_I_AFNI01000333                : -------------------------------------------------------------------------------- :    -
NKorean_AFNJ01000531                     : TGCAAACGCCGCAAATGNNN-------AAATGCAAACCTCGCAAACGCAAACGCCGCAAACGCAAACGCCGCAAACGCAA : 1893
India-VII_AFBK01000586_AFBK01000587      : TGCAAACGCCGCAAATNNGAACCTCGCAAATGCAAACCTCGCAAACGCAAACGCCGCA---------------------- : 1879

                                                    *      2100         *      2120         *      2140         *      2160
P.coatneyi_CM002856.1                    : -------------------------------------------------------------------------------- :    -
Pknowlesi_NC_011906.1                    : -------------------------------------------------------------------------------- :    -
Pinui_NW_008481888.1                     : -------------------------------------------------------------------------------- :    -
Pfragile_NW_012192586.1                  : -------------------------------------------------------------------------------- :    -
Pcynomolgi_BAEJ01000249                  : -------------------------------------------------------------------------------- :    -
Brazil-I_AFMK01000508.1_A                : -------------------------------------------------------------------------------- :    -
VCG-I                                    : ACGCCGCAAACGCAAACGCCGCAAACGCAAACGCCGCAAATGCAAACCTCGCAAATGCAAACCTCGCAAATGCAAACCTC : 1983
Sal-1_PVX_088910                         : ACGCCGCAAACGCAAACGCCGCTAATGCAAACGCCGCAAATGCAAACCTCGCAAATGCAAACCTCGCAAATGCAAACCTC : 1983
Mauritania_I_AFNI01000333                : -------------------------------------------------------------------------------- :    -
NKorean_AFNJ01000531                     : ACGCCGCAAACGCAAACGCCGCTAATGCAAACGCCGCAAATGGG------------------------------------ : 1937
India-VII_AFBK01000586_AFBK01000587      : --------------------------------------AATGGG------------------------------------ : 1885
```

```
                                                  *      2180         *      2200         *      2220         *      2240
P.coatneyi_CM002856.1                  : -------------------------------------------------------------------------------- :    -
Pknowlesi_NC_011906.1                  : -------------------------------------------------------------------------------- :    -
Pinui_NW_008481888.1                   : -------------------------------------------------------------------------------- :    -
Pfragile_NW_012192586.1                : ------------------------------------------------------------------------------AG : 1808
Pcynomolgi_BAEJ01000249                : ------------------------------------------------------AACAACGCAAATGGGAACAACGCAAA : 1907
Brazil-I_AFMK01000508.1_A              : GCAAATGCAAACCTCGCAAATGCAAACCTCGCAAATGCAAACCTCGCAAACGCAAACGCCGCAAACGCAAACGCCGCTAA : 1955
VCG-I                                  : GCAAATGCAAACCTCGCAAATGCAAACCTCGCAAATGCAAACCTCGCAAACGCAAACGCCGCAAACGCAAACGCCGCTAA : 2063
Sal-1_PVX_088910                       : GCAAATGCAAACCTCGCAAATGCAAACCTCGCAAATGCAAACCTCGCAAACGCAAACGCCGCAAACGCAAACGCCGCTAA : 2063
Mauritania_I_AFNI01000333              : ---------------------------------------------GCAAACGCAAACGCCGCAAACGCAAACGCCGCTAA : 1817
NKorean_AFNJ01000531                   : -------------------------------------------------------------------------------- :    -
India-VII_AFBK01000586_AFBK01000587    : -------------------------------------------------------------------------------- :    -

                                                  *      2260         *      2280         *      2300         *      2320
P.coatneyi_CM002856.1                  : -------------------AACGCCCAAAATGGAAACGGCGCAAATGTAAATGCTCAAAATGAAAACGCCCCAAATGGAA : 1918
Pknowlesi_NC_011906.1                  : -------------------AACAACATGAATGGA------------------------------------------GGTA : 1951
Pinui_NW_008481888.1                   : ----------------------------------------------------------------AACGCCGCAAATGGAA : 1891
Pfragile_NW_012192586.1                : CGGACATGCGGCAAATGAC---------------------------------------------AACGCACATGCTGCAA : 1843
Pcynomolgi_BAEJ01000249                : TGGAAACAACGCAAATGGA---------------------------------------------AACGCCGCAAATGGAA : 1942
Brazil-I_AFMK01000508.1_A              : TGCAAACGCCGCAAATGGG---------------------------------------------AACGCCCCAAATAGCA : 1990
VCG-I                                  : TGCAAACGCCGCAAATGGG---------------------------------------------AACGCCCCAAATAGCA : 2098
Sal-1_PVX_088910                       : TGCAAACGCCGCAAATGGG---------------------------------------------AACGCCCCAAATAGCA : 2098
Mauritania_I_AFNI01000333              : TGCAAACGCCGCAAATGGG---------------------------------------------AACGCCCCAAATAGCA : 1852
NKorean_AFNJ01000531                   : ----------------------------------------------------------------AACGCCCCAAATAGCA : 1953
India-VII_AFBK01000586_AFBK01000587    : ----------------------------------------------------------------AACGCCCCAAATAGCA : 1901
                                                                                                         aacgcc caaat g A

                                                  *      2340         *      2360         *      2380         *      2400
P.coatneyi_CM002856.1                  : ACAACGGAAGCGCCTCCCCCCTCATCGTAGTGGTAGGGGACGAGCTCGGAGAAAAAACCGACGATATCATCAAAAAAAT : 1998
Pknowlesi_NC_011906.1                  : ACAACGGAAGCGCCTCCTCCCTCATCGTAGTAGTAAGGGACGATCTCGCTGAAAAAACTGATGACATCATCAAAAATAAT : 2031
Pinui_NW_008481888.1                   : ACAACGGAAGCGGCTCCCCCCTCCTCGTAGTGGTAGGGGCCGATCTCGGCGAAAAAACCGAAGACATCATCAAGAACAAT : 1971
Pfragile_NW_012192586.1                : ATGACNNN------------------------------------------------------------------------ : 1848
Pcynomolgi_BAEJ01000249                : ACAACGGAAACGGATCCCCCCTCATCGCAGTGGTAGGGGCCGATCTCGGCGAAAAAACTGACGACATCATCAAGAACAAT : 2022
Brazil-I_AFMK01000508.1_A              : ACAACGGAAGCGGATCCCCCCTCATCGTAGTGGTAGGGGCCGATCTCGGCGAAAAAACCGAGGACATCATCAAGAACAAC : 2070
VCG-I                                  : ACAACGGAAGCGGATCCCCCCTCATCGTAGTGGTAGGGGCCGATCTCGGCGAAAAAACCGAGGACATCATCAAGAACAAC : 2178
Sal-1_PVX_088910                       : ACAACGGAAGCGGATCCCCCCTCATCGTAGTGGTAGGGGCCGATCTCGGCGAAAAAACCGAGGACATCATCAAGAACAAC : 2178
Mauritania_I_AFNI01000333              : ACAACGGAAGCGGATCCCCCCTCATCGTAGTGGTAGGGGCCGATCTCGGCGAAAAAACCGAGGACATCATCAAGAACAAC : 1932
NKorean_AFNJ01000531                   : ACAACGGAAGCGGATCCCCCCTCATCGTAGTGGTAGGGGCCGATCTCGGCGAAAAAACCGAGGACATCATCAAGAACAAC : 2033
India-VII_AFBK01000586_AFBK01000587    : ACAACGGAAGCGGATCCCCCCTCATCGTAGTGGTAGGGGCCGATCTTGGCGAAAAAACCGAGGACATCATCAAGAACAAC : 1981
                                         AcaACggaagcg  tcccccctcatcgtagtggtagggg cgatctcgg gaaaaaac ga gacatcatcaa aa aa
```

```
                                                   *        2420         *        2440         *        2460         *        2480
P.coatneyi_CM002856.1                          : GTGGACGTGGAAGCCTTGACAGCCGACGTGGAACAAGCCTTCAAAAGCTTCGAATACCAAAGTGGAAGTTTCTCTGCAAA : 2078
Pknowlesi_NC_011906.1                          : GTGGACCTGGAATCCTTAAAAGCCGACGTGGAACAAGCCTTTAGAAACTTCGAATACCAAAGTGGAAGTTTCTCGGCAAA : 2111
Pinui_NW_008481888.1                           : GTGGACGTGGCAACCTTGACAGAAGGCGTGGAACAAGCCTTTAAAAACCTCTCATTGGAAGGTGGAAGTTTCTCGGCAAA : 2051
Pfragile_NW_012192586.1                        : -------------------------------------------------------------------------------- :    -
Pcynomolgi_BAEJ01000249                        : GTGGACGTGGCAGCCTTGGCAGCCGACGTGGAACAAGCCTTTAGAAACCTCGAATTGAAAAGTGGAAGCTTCTCGGCAAA : 2102
Brazil-I_AFMK01000508.1_A                      : GTGGACGTGGCAGCCTTGACAGCCGACGTGGAACAAGCCTTTAAAAACCTCGAATTGCAGAGTGGAAGCTTCTCGGCAAA : 2150
VCG-I                                          : GTGGACGTGGCAGCCTTGACAGCCGACGTGGAACAAGCCTTTAAAAACCTCGAATTGCAGAGTGGAAGCTTCTCGGCAAA : 2258
Sal-1_PVX_088910                               : GTGGACGTGGCAGCCTTGACAGCCGACGTGGAACAAGCCTTTAAAAACCTCGAATTGCAGAGTGGAAGCTTCTCGGCAAA : 2258
Mauritania_I_AFNI01000333                      : GTGGACGTGGCAGCCTTGACAGCCGACGTGGAACAAGCCTTTAAAAACCTCGAATTGCAGAGTGGAAGCTTCTCGGCAAA : 2012
NKorean_AFNJ01000531                           : GTGGACGTGGCAGCCTTGACAGCCGACGTGGAACAAGCCTTTAAAAACCTCGAATTGCAGAGTGGAAGCTTCTCGGCAAA : 2113
India-VII_AFBK01000586_AFBK01000587            : GTGGACGTGGCAGCCTTGACAGCCGACGTGGAACAAGCCTTTAAAAACCTCGAATTGCAGAGTGGAAGCTTCTCGGCAAA : 2061
                                                 gtggacgtgg a ccttgacagccgacgtggaacaagccttta aaac tcgaat   a agtggaag ttctcggcaaa

                                                   *        2500         *        2520         *
P.coatneyi_CM002856.1                          : CTTATCTCACGCCCTGGTTCTCCTCTCCTCCATTGCGTTGCTCTTATTCATTTGT : 2133
Pknowlesi_NC_011906.1                          : CTTATCTCACGCCCTGGTTCTCCTATCCTCCATTGCGTTAGTCCTATTCATTTGT : 2166
Pinui_NW_008481888.1                           : CTTATCGCACGCGCTGGTTCTCCTCTCTTCCATTGCTTTACTCCTATTCATTTGT : 2106
Pfragile_NW_012192586.1                        : ------------------------------------------------------- :    -
Pcynomolgi_BAEJ01000249                        : CTTATCGCACGCGCTGGTTCTCCTCTCTTCCATTGCGTTACTCCTATTCATTTTT : 2157
Brazil-I_AFMK01000508.1_A                      : CTTATCGCACGCGCTGGTTCTCCTCTCCTCCATTGCGTTGCTCCTATTCATTTTT : 2205
VCG-I                                          : CTTATCGCACGCGCTGGTTCTCCTCTCCTCCATTGCGTTGCTCCTATTCATTTTT : 2313
Sal-1_PVX_088910                               : CTTATCGCACGCGCTGGTTCTCCTCTCCTCCATTGCGTTGCTCCTATTCATTTTT : 2313
Mauritania_I_AFNI01000333                      : CTTATCGCACGCGCTGGTTCTCCTCTCCTCCATTGCGTTGCTCCTATTCATTTTT : 2067
NKorean_AFNJ01000531                           : CTTATCGCACGCGCTGGTTCTCCTCTCCTCCATTGCGTTGCTCCTATTCATTTTT : 2168
India-VII_AFBK01000586_AFBK01000587            : CTTATCGCACGCGCTGGTTCTCCTCTCCTCCATTGCGTTGCTCCTATTCATTTTT : 2116
                                                 cttatc cacgc ctggttctcctctc tccattgcgtt ctcctattcattt t
```

b. Deduced amino acid alignment.

```
                                               *        20         *        40         *        60         *        80
P.coatneyi_CM002856.1               : MKCNGSLLVLLSAILSATNALIRNGNNPQALVPEKSDDSSGGQNKPSGDNQDTCEIQKMAEEMMEKMVNEKDVFTSIMEPL :  81
Pknowlesi_NC_011906.1               : MKCNGSLLVLLSAILSATNALIRNGNNPQALVPDNSGDPNAGQNKTSSDNQDTCEIQKMAEEMMEKMMKEKDVFSSIMEPL :  81
Pinui_NW_008481888.1                : MKCNTALLVLLSAILSAANALIRNGNTTQALVPEKSNDPSGGQNNPSGDNRETCEIQQMAQEMMEKMMKEKDVFSSIMEPL :  81
Pfragile_NW_012192586.1             : MKYNATLLVLLSAILSAGKALIRNGNNTQALVPENGGDPSGGQNNPSGENQDTCEVQKMAEEMMGKMMKEKDVFSSIMEPL :  81
Pcynomolgi_BAEJ01000249             : MKCNAALLVLLSALLSASNALIRNGNNTQALVPEKSGDPSGGQNNPSGDNQDTCEIQKMAEEMMEKMMKEKDMFSSIMEPL :  81
Brazil-I_AFMK01000508.1_A           : MKCNASLLVLLSALLSAANALIRNGNNPQALVPEKGADPSGGQNNRSGENQDTCEIQKMAEEMMEKMMKEKDVFSSIMEPL :  81
VCG-I                               : MKCNASLLVLLSALLSAANALIRNGNNPQALVPEKGADPSGGQNNRSGENQDTCEIQKMAEEMMEKMMKEKDVFSSIMEPL :  81
Sal-1_PVX_088910                    : MKCNASLLVLLSALLSAANALIRNGNNPQALVPEKGADPSGGQNNRSGENQDTCEIQKMAEEMMEKMMKEKDVFSSIMEPL :  81
Mauritania_I_AFNI01000333           : MKCNASLLVLLSALLSAANALIRNGNNPQALVPEKGADPSGGQNNRSGENQDTCEIQKMAEEMMEKMMKEKDVFSSIMEPL :  81
N.Korean_AFNJ01000531               : MKCNASLLVLLSALLSAANALIRNGNNPQALVPEKGADPSGGQNNRSGENQDTCEIQKMAEEMMEKMMKEKDVFSSIMEPL :  81
India-VII_AFBK01000586_AFBK01000587 : MKCNASLLVLLSALLSAANALIRNGNNPQALVPEKGADPSGGQNNRSGENQDTCEIQKMAEEMMEKMMKEKDVFSSIMEPL :  81
                                      MKcN  LLVLLSA6LSA nALIRNGNn QALVPek  DpsgGQNn Sg NqdTCE6QkMA2EMMeKM6kEKD6F3SIMEPL

                                              *       100         *       120         *       140         *       160
P.coatneyi_CM002856.1               : QSKLTDDHLCSKLKYTNVCLHEKDKTPLTFPCTSPEYEKLIQQFTYQKLCNSKVAFSNVLLKSFIDKKNEENTFNAIIQNY : 162
Pknowlesi_NC_011906.1               : QSKLTDDRLCSKMKYTNICLHEKDKTPLTFPCTNPQYEQLIQQFTYKKLCNSKVAFSNVLLKSFIDKKNEENTFNAIIQNY : 162
Pinui_NW_008481888.1                : QSKLADDHLCSEVKYKNICLQEKDNNSLTFPCTSPEYEQLIHEFTYKKLCNSKVAFSNVLLKSFIDKKNEENTFNTIIENY : 162
Pfragile_NW_012192586.1             : QSKLSDNHLCSTVKYTNICLHEKDKTPLTFPCTSPQYEQLIHHFTYKKLCNSQVAFNNVLLKSFINKNNEENTFNTIIQNY : 162
Pcynomolgi_BAEJ01000249             : QSKLPDDHLCSKLKYTNICLQEKDKTPLTLPCTSPQYEQLIQHFTYKKLCNSQVAFSNVLLKSFINKKNEENTFNTIIQNY : 162
Brazil-I_AFMK01000508.1_A           : QSKLTDDHLCSKMKYTNICLHEKDKTPLTFPCTSPQYEQLIHRFTYKKLCNSKVAFSNVLLKSFIDKKNEENTFNTIIQNY : 162
VCG-I                               : QSKLTDDHLCSKMKYTNICLHEKDKTPLTFPCTSPQYEQLIHRFTYKKLCNSKVAFSNVLLKSFIDKKNEENTFNTIIQNY : 162
Sal-1_PVX_088910                    : QSKLTDDHLCSKMKYTNICLHEKDKTPLTFPCTSPQYEQLIHRFTYKKLCNSKVAFSNVLLKSFIDKKNEENTFNTIIQNY : 162
Mauritania_I_AFNI01000333           : QSKLTDDHLCSKMKYTNICLHEKDKTPLTFPCTSPQYEQLIHRFTYKKLCNSKVAFSNVLLKSFIDKKNEENTFNTIIQNY : 162
N.Korean_AFNJ01000531               : QSKLTDDHLCSKMKYTNICLHEKDKTPLTFPCTSPQYEQLIHRFTYKKLCNSKVAFSNVLLKSFIDKKNEENTFNTIIQNY : 162
India-VII_AFBK01000586_AFBK01000587 : QSKLTDDHLCSKMKYTNICLHEKDKTPLTFPCTSPQYEQLIHRFTYKKLCNSKVAFSNVLLKSFIDKKNEENTFNTIIQNY : 162
                                      QSKL D1hLCSk6KYtN6CLhEKDktpLTfPCTsP2YEqLI  FTYkKLCNSkVAFsNVLLKSFI1KkNEENTFNtII2NY

                                             *       180         *       200         *       220         *       240
P.coatneyi_CM002856.1               : KVLSTCIDEDLKDIYNASIELFSDIRSSVTEITEKLWSKNMIDVLKTREQAIAGILCELRNGNNSPLVSNSLSYENFGILK : 243
Pknowlesi_NC_011906.1               : KVLSTCIDEDLKDIYNASIELFSDLRTSVREITEKLWSKNMIEVLKTREQAIAGILCELRNGNNSTLVSNSLSYENFGILK : 243
Pinui_NW_008481888.1                : KVLSTCIDADLKDIYSASIQLFSDLRSAVTEISERLWSKNMIDVLKTREETITGILCELRKGNNSTLVSNSFSYDNFGILK : 243
Pfragile_NW_012192586.1             : KILSTCIDEDLKDIYDASIQLFSDIRVSVTEITEKLWSKNMIDVLKTREQTIAGILCELRNGNKSTLVSNSLSYENFGILK : 243
Pcynomolgi_BAEJ01000249             : KLLSTCIDEDLKDIYNASIDLFSDIRTSVTEITEKLWSKNMIEVLKAREQTIAGILCELRNGNNSTLVSNSLSYENFGILK : 243
Brazil-I_AFMK01000508.1_A           : KVLSTCIDDDLKDIYNASIELFSDIRTSVTEITEKLWSKNMIEVLKTREQTIAGILCELRNGNNSPLVSNSFSYENFGILK : 243
VCG-I                               : KVLSTCIDDDLKDIYNASIELFSDIRTSVTEITEKLWSKNMIEVLKTREQTIAGILCELRNGNNSPLVSNSFSYENFGILK : 243
Sal-1_PVX_088910                    : KVLSTCIDDDLKDIYNASIELFSDIRTSVTEITEKLWSKNMIEVLKTREQTIAGILCELRNGNNSPLVSNSFSYENFGILK : 243
Mauritania_I_AFNI01000333           : KVLSTCIDDDLKDIYNASIELFSDIRTSVTEITEKLWSKNMIEVLKTREQTIAGILCELRNGNNSPLVSNSFSYENFGILK : 243
N.Korean_AFNJ01000531               : KVLSTCIDDDLKDIYNASIELFSDIRTSVTEITEKLWSKNMIEVLKTREQTIAGILCELRNGNNSPLVSNSFSYENFGILK : 243
India-VII_AFBK01000586_AFBK01000587 : KVLSTCIDDDLKDIYNASIELFSDIRTSVTEITEKLWSKNMIEVLKTREQTIAGILCELRNGNNSPLVSNSFSYENFGILK : 243
                                      K6LSTCID DLKDIY ASI LFSD6R sVtEI3E4LWSKNMI VLKtRE2tIaGILCELRnGNnS LVSNS SYeNFGILK
```

```
                                                    *         260         *         280         *         300         *         320
P.coatneyi_CM002856.1                        : VNYEALMNQAYKAFSDYYSYFPAFAIKLLEKGGFVERLVAIHESLTNYRTKNILKKINEKSKNEVLNNEDIMHSLSSYKHH : 324
Pknowlesi_NC_011906.1                        : VNYEGLINQAYKAFSDYYSYFPAFAIRLLEKDGLVERLVAIHESLTNYRTRNILKKINEKSKNEVLNNEDIMHSLSSYKHH : 324
Pinui_NW_008481888.1                         : VNYEGLLNQAYVAFSDYYSFFPLFAMRLLENGGLVERLVAIHEKLTNYRTRNILKKINDKSKNEVLNNEDIMHSLSSYKHH : 324
Pfragile_NW_012192586.1                      : VNYEGLMNQAYKAFSDYYSYFPDFAIKLLEKGGLVERLVAIHESLTNYRTKNILQKINEKSKNEVLNNEDIMHSLSSYKHR : 324
Pcynomolgi_BAEJ01000249                      : VNYEELLNQAYAAFSDYYSYFPAFAIKLLEKGGLVERLVAIHESLTNYRTRNILKKINEKSKNEVLNNGDIMHSLSSYKHH : 324
Brazil-I_AFMK01000508.1_A                    : VNYEGLLNQAYAAFSDYYSYFPAFAISMLEKGGLVDRLVAIHESLTNYRTRNILKKINEKSKNEVLNNEEIMHSLSSYKHH : 324
VCG-I                                        : VNYEGLLNQAYAAFSDYYSYFPAFAISMLEKGGLVDRLVAIHESLTNYRTRNILKKINEKSKNEVLNNEEIMHSLSSYKHH : 324
Sal-1_PVX_088910                             : VNYEGLLNQAYAAFSDYYSYFPAFAISMLEKGGLVDRLVAIHESLTNYRTRNILKKINEKSKNEVLNNEEIMHSLSSYKHH : 324
Mauritania_I_AFNI01000333                    : VNYEGLLNQAYAAFSDYYSYFPAFAISMLEKGGLVDRLVAIHESLTNYRTRNILKKINEKSKNEVLNNEEIMHSLSSYKHH : 324
N.Korean_AFNJ01000531                        : VNYEGLLNQAYAAFSDYYSYFPAFAISMLEKGGLVDRLVAIHESLTNYRTRNILKKINEKSKNEVLNNEEIMHSLSSYKHH : 324
India-VII_AFBK01000586_AFBK01000587          : VNYEGLLNQAYAAFSDYYSYFPAFAISMLEKGGLVDRLVAIHESLTNYRTRNILKKINEKSKNEVLNNEEIMHSLSSYKHH : 324
                                               VNYEgL6NQAY AFSDYYS5FPaFA6 6LEkgGlV RLVAIHEsLTNYRT4NILkKINeKSKNEVLNNe IMHSLSSYKHh

                                                    *         340         *         360         *         380         *         400
P.coatneyi_CM002856.1                        : AGGTRGSFVQSRGVSQVTQGVLSVDEKGDPSSTAG-GNQSANMATAAPN-------------TVPATNTAAATTATTTNTA : 391
Pknowlesi_NC_011906.1                        : AGGTRGSFMQYRGVGQLSKGGLSVDEKGGQQIASAVGNQSANMVAPAPKDSSPTM-------AAPSTDTVTNNMATTTTAP : 398
Pinui_NW_008481888.1                         : ATGTRGSFLQSRGVRQVTNSNVSVDQKGDQTATAG-GNESENVPAAVPKDDAPTRVASPGPAAFPNTVASNPTAVSNATAA : 404
Pfragile_NW_012192586.1                      : AGSTRGSFLQSRGAHNVTKVDVKVDE----TSTTG-GNQSANMAAAAPNNSGPTTAAAANSAASPNTAATTNTAATTS--- : 397
Pcynomolgi_BAEJ01000249                      : AGGTRGSFLQSRVAREVRQGDVSVDEKGDQTATTG-GNQSANMAAAAPKDASPTT-AASNAASPDTTAANTAAANTAAAN : 403
Brazil-I_AFMK01000508.1_A                    : AGGTRGAFLQSRDVREVTQGDVSVDEKGDRATTAG-GNQSASVAAAAPKDAGPTV-------AAPNTAATLKTA------- : 390
VCG-I                                        : AGGTRGAFLQSRDVREVTQGDVSVDEKGDRATTAG-GNQSASVAAAAPKDAGPTV-------AAPNTAATLKTA------- : 390
Sal-1_PVX_088910                             : AGGTRGAFLQSRDVREVTQGDVSVDEKGDRATTAG-GNQSASVAAAAPKDAGPTV-------AAPNTAATLKTA------- : 390
Mauritania_I_AFNI01000333                    : AGGTRGAFLQSRDVREVTQGDVSVDEKGDRATTAG-GNQSASVAAAAPKDAGPTV-------AAPNTGATLKTA------- : 390
N.Korean_AFNJ01000531                        : AGGTRGAFLQSRDVREVTQGDVSVDEKGDRATTAG-GNQSASVAAAAPKDAGPTV-------AAPNTAATLKTA------- : 390
India-VII_AFBK01000586_AFBK01000587          : AGGTRGAFLQSRDVREVTQGDVSVDEKGDRATTAG-GNQSASVAAAAPKDAGPTV-------AAPNTAATLKTA------- : 390
                                               AggTRG F6QsR v  6  g 6sVD2kgd   t g GN2Sa 6aaaaPk   pt        a P T a   ta

                                                  *         420         *         440         *         460         *         480
P.coatneyi_CM002856.1                        : TNTNTA------TNTNTAT-NTNSAATTNTAA------TTNTAATTNTTATTSVATSPLNT-------PLYGTSSSRTKDV : 452
Pknowlesi_NC_011906.1                        : ATANMAAPSTNTATANMAA-PSTNTATANMAAPSTDTVTTNMATTTTVPATANTAASP-DTNTDSSTYPLYGTESSKTKDV : 477
Pinui_NW_008481888.1                         : SNATAASNATAASNATD---ASNATAASNPAA-----------------PPNTAA-TSQ-LSN-------PLYGISSLNTKDV : 458
Pfragile_NW_012192586.1                      : --------------------ASNDTNASPHTN-----------------TSNEGASTSP-LSN-------PLYGTRYLKTKDV : 435
Pcynomolgi_BAEJ01000249                      : TAAAPNTASASNAAATPNPAAAPNTAATPNSA-----------------TPNAAA-TSP-LST-------PLYDTSSLKTKDV : 460
Brazil-I_AFMK01000508.1_A                    : --------------------ASPNAAATNTAA-----------------PPNMGA-TSP-LSN-------PLYGTSSLQPKDV : 427
VCG-I                                        : --------------------ASPNAAATNTAA-----------------PPNMGA-TSP-LSN-------PLYGTSSLQPKDV : 427
Sal-1_PVX_088910                             : --------------------ASPNAAATNTAA-----------------PPNMGA-TSP-LSN-------PLYGTSSLQPKDV : 427
Mauritania_I_AFNI01000333                    : --------------------ASPNAAATNTAA-----------------PPNMGA-TSP-LSN-------PLYGTSSLQPKDV : 427
N.Korean_AFNJ01000531                        : --------------------ASPNAAATNTAA-----------------PPNMGA-TSP-LSN-------PLYGTSSLQPKDV : 427
India-VII_AFBK01000586_AFBK01000587          : --------------------ASPNAAATNTAA-----------------PPNMGA-TSP-LSN-------PLYGTSSLQPKDV : 427
                                                                   a    aa n aa                  n  a tSp l3n       PLYgtssl  KDV
```

```
                                       *        500         *        520         *        540         *        560
P.coatneyi_CM002856.1               : VVLVRDLLKNTNIIKFEKNEPTSQIDDEGIKKLIESSFFDLSDNTMLMRLLIKPQAAILFIIQSFIMMTPSPTRDAKMYCK : 533
Pknowlesi_NC_011906.1               : VVLVRDLLKDTNIIKFEKNEPTSQIDDEGIKKLIESSFFDLSDNTMLMRLIIKPQASILFIIQSFIMMTPSPTRDARMYCK : 558
Pinui_NW_008481888.1                : VVLVRDLLKNTNIIKFDKDEPTSQMDDEGIKKLIESSFFDLSDNTMLMQLLIKPQAAMLFIIQSFIMMTPSPTRDAKTYCN : 539
Pfragile_NW_012192586.1             : VVLIRDLLKNTNIIKFEKDEPTGQIDHEGIKKLIESSFFDLSDNTMLMRLLIKPQAAILFIIQSFIMMTPSPTRDAKIYCK : 516
Pcynomolgi_BAEJ01000249             : VVLVRDLLKNTNIIKFEKDEPTSQIDEEGIKKLIESSFFDLSDNTMLMRLLIKPQAAILFIIQSFIMMTPSPTRDAKIYCK : 541
Brazil-I_AFMK01000508.1_A           : AVLVRDLLKNTNIIKFENNEPTSQMDDEEIKKLIESSFFDLSDNTMLMRLLIKPQAAILLIIESFIMMTPSPTRDAKTYCK : 508
VCG-I                               : AVLVRDLLKNTNIIKFENNEPTSQMDDEEIKKLIESSFFDLSDNTMLMRLLIKPQAAILLIIESFIMMTPSPTRDAKTYCK : 508
Sal-1_PVX_088910                    : AVLVRDLLKNTNIIKFENNEPTSQMDDEEIKKLIESSFFDLSDNTMLMRLLIKPQAAILLIIESFIMMTPSPTRDAKTYCK : 508
Mauritania_I_AFNI01000333           : AVLVRDLLKNTNIIKFENNEPTSQMDDEEIKKLIESSFFDLSDNTMLMRLLIKPQAAILLIIESFIMMTPSPTRDAKTYCK : 508
N.Korean_AFNJ01000531               : AVLVRDLLKNTNIIKFENNEPTSQMDDEEIKKLIESSFFDLSDNTMLMRLLIKPQAAILLIIESFIMMTPSPTRDAKTYCK : 508
India-VII_AFBK01000586_AFBK01000587 : AVLVRDLLKNTNIIKFENNEPTSQMDDEEIKKLIESSFFDLSDNTMLMRLLIKPQAAILLIIESFIMMTPSPTRDAKTYCK : 508
                                       VL6RDLLK1TNIIKFe 1EPTsQ6DdE IKKLIESSFFDLSDNTMLMrL6IKPQAa6L II2SFIMMTPSPTRDA4 YCk

                                        *        580         *        600         *        620         *        640
P.coatneyi_CM002856.1               : KKLVNGQLIENSDLKAATEEEDMITEFSSKYNLFYERLKLEELREIEQNRKALKNSKGSLSVLQVGNSQNGPDGKEANGSG : 614
Pknowlesi_NC_011906.1               : KKLVNGQLIENNDLKAETEEEDMINEFSSKYNLFYERLKMEELREIEQDRKSLKNSKGNLSVLEVRNSQNGPDGKEVNGSG : 639
Pinui_NW_008481888.1                : KSLVNGKLIETSDIKASTEEEDLITEFSSKYNLFYEKLKMEELREIEENKKAIKNSKGSLSVLEVANSQNSPDGKRVNGSG : 620
Pfragile_NW_012192586.1             : KNLVNGQLVQTSDFSDAHEEEDLINEFSSKYNLFYERLKLEELREIEQNRKALKNAKGSLSVLQVSNSQNGTHGNGVNQGG : 597
Pcynomolgi_BAEJ01000249             : KVLVNGQLVETSDLSAATEEEDLINEFSSKYNLFYERLKLEELREIEQNRKALKNSKGSLSVLEVGNSQNGPDGSGNNGSG : 622
Brazil-I_AFMK01000508.1_A           : KALVNGQLIETSDLNAATEEDDLINEFSSRYNLFYERLKLEELREIEQNRKALKNSKGTLSVLEVANSQNAPDGKGVNGSG : 589
VCG-I                               : KALVNGQLIETSDLNAATEEDDLINEFSSRYNLFYERLKLEELREIEQNRKALKNSKGTLSVLEVANSQNAPDGKGVNGSG : 589
Sal-1_PVX_088910                    : KALVNGQLIETSDLNAATEEDDLINEFSSRYNLFYERLKLEELREIEQNRKALKNSKGTLSVLEVANSQNAPDGKGVNGSG : 589
Mauritania_I_AFNI01000333           : KALVNGQLIETSDLNATTEEDDLINEFSSRYNLFYERLKLEELREIEQNRKALKNSKGTLSVLEVANSQNAPDGKGVNGSG : 589
N.Korean_AFNJ01000531               : KALVNGQLIETSDLNAATEEDDLINEFSSRYNLFYERLKLEELREIEQNRKALKNSKGTLSVLEVANSQNAPDGKGVNGSG : 589
India-VII_AFBK01000586_AFBK01000587 : KALVNGQLIETSDLNAATEEDDLINEFSSRYNLFYERLKLEELREIEQNRKALKNSKGTLSVLEVANSQNAPDGKGVNGSG : 589
                                      K LVNGqL62tsD  a tEE D6InEFSS4YNLFYE4LK6EELREIE214Ka6KNsKG LSVL2V NSQN pdGk vNgsG

                                        *        660         *        680         *        700         *        720
P.coatneyi_CM002856.1               : NAANG--------------------------------------------------------------------------- : 619
Pknowlesi_NC_011906.1               : DAANG--------------------------------------------------------------------------- : 644
Pinui_NW_008481888.1                : NDANG--------------------------------------------------------------------------- : 625
Pfragile_NW_012192586.1             : HAANE--------------------------------------------------------------------------- : 602
Pcynomolgi_BAEJ01000249             : NAANG--------------------------------------------------------------------------- : 627
Brazil-I_AFMK01000508.1_A           : NAANANAANANAANANAANANAANGNLANANLANAN-----------------------------------ANANLANAN : 634
VCG-I                               : NAANANAANANAANANAANANAANGNLANANLANANAANADAANANAANANAANANAANANLANANLANANLANANLANAN : 670
Sal-1_PVX_088910                    : NAANANAANANAANANAANANAANGNLANANLANANAANADAANANAANANAANANAANANLANANLANANLANANLANAN : 670
Mauritania_I_AFNI01000333           : NAANA--------------------------------------------------------------------------- : 594
N.Korean_AFNJ01000531               : NAANANAANANAANANAANANAANX--XNANLANANAANANAANANAANANAANANAANG-------------------- : 645
India-VII_AFBK01000586_AFBK01000587 : NAANANAANANAANANAANANAANXNLANANLANANAA--------------------NG-------------------- : 628
                                       aAN
```

```
                                             *       740         *       760         *       780         *       800         *
P.coatneyi_CM002856.1               : ------------------------NAQNGNGANVNAQNENAPNGNNGSASPLIVVVGDELGEKTDDIIKKNVDVEALTADV : 676
Pknowlesi_NC_011906.1               : ------------------------NNMNG--------------GNNGSASSLIVVVRDDLAEKTDDIIKNNVDLESLKADV : 687
Pinui_NW_008481888.1                : ---------------------------------------NAANGNNGSGSPLLVVVGADLGEKTEDIIKNNVDVATLTEGV : 667
Pfragile_NW_012192586.1             : -----------------SGHAAND---------------NAHAAND----------------------------------- : 616
Pcynomolgi_BAEJ01000249             : ---------NNANGNNANGNNANG---------------NAANGNNGNGSPLIAVVGADLGEKTDDIIKNNVDVAALAADV : 684
Brazil-I_AFMK01000508.1_A           : LANANLANANAANANAANANAANG---------------NAPNSNNGSGSPLIVVVGADLGEKTEDIIKNNVDVAALTADV : 700
VCG-I                               : LANANLANANAANANAANANAANG---------------NAPNSNNGSGSPLIVVVGADLGEKTEDIIKNNVDVAALTADV : 736
Sal-1_PVX_088910                    : LANANLANANAANANAANANAANG---------------NAPNSNNGSGSPLIVVVGADLGEKTEDIIKNNVDVAALTADV : 736
Mauritania_I_AFNI01000333           : ------ANANAANANAANANAANG---------------NAPNSNNGSGSPLIVVVGADLGEKTEDIIKNNVDVAALTADV : 654
N.Korean_AFNJ01000531               : ---------------------------------------NAPNSNNGSGSPLIVVVGADLGEKTEDIIKNNVDVAALTADV : 687
India-VII_AFBK01000586_AFBK01000587 : ---------------------------------------NAPNSNNGSGSPLIVVVGADLGEKTEDIIKNNVDVAALTADV : 670
                                                                             na n N1gs spl vvvg dlgekt diiknnvd   l adv

                                              820         *       840
P.coatneyi_CM002856.1               : EQAFKSFEYQSGSFSANLSHALVLLSSIALLLFIC : 711
Pknowlesi_NC_011906.1               : EQAFRNFEYQSGSFSANLSHALVLLSSIALVLFIC : 722
Pinui_NW_008481888.1                : EQAFKNLSLEGGSFSANLSHALVLLSSIALLLFIC : 702
Pfragile_NW_012192586.1             : ----------------------------------- :   -
Pcynomolgi_BAEJ01000249             : EQAFRNLELKSGSFSANLSHALVLLSSIALLLFIF : 719
Brazil-I_AFMK01000508.1_A           : EQAFKNLELQSGSFSANLSHALVLLSSIALLLFIF : 735
VCG-I                               : EQAFKNLELQSGSFSANLSHALVLLSSIALLLFIF : 771
Sal-1_PVX_088910                    : EQAFKNLELQSGSFSANLSHALVLLSSIALLLFIF : 771
Mauritania_I_AFNI01000333           : EQAFKNLELQSGSFSANLSHALVLLSSIALLLFIF : 689
N.Korean_AFNJ01000531               : EQAFKNLELQSGSFSANLSHALVLLSSIALLLFIF : 722
India-VII_AFBK01000586_AFBK01000587 : EQAFKNLELQSGSFSANLSHALVLLSSIALLLFIF : 705
                                      eqaf n e  sgsfsanlshalvllssial lfi
```
