# Supplementary figures and images for: PvGAMA reticulocyte binding activity: predicting conserved functional regions by natural selection analysis
Source: Parasit Vectors. 2017 May 19;10:251. doi: 10.1186/s13071-017-2183-8 (PMC5438544; doi:10.1186/s13071-017-2183-8)

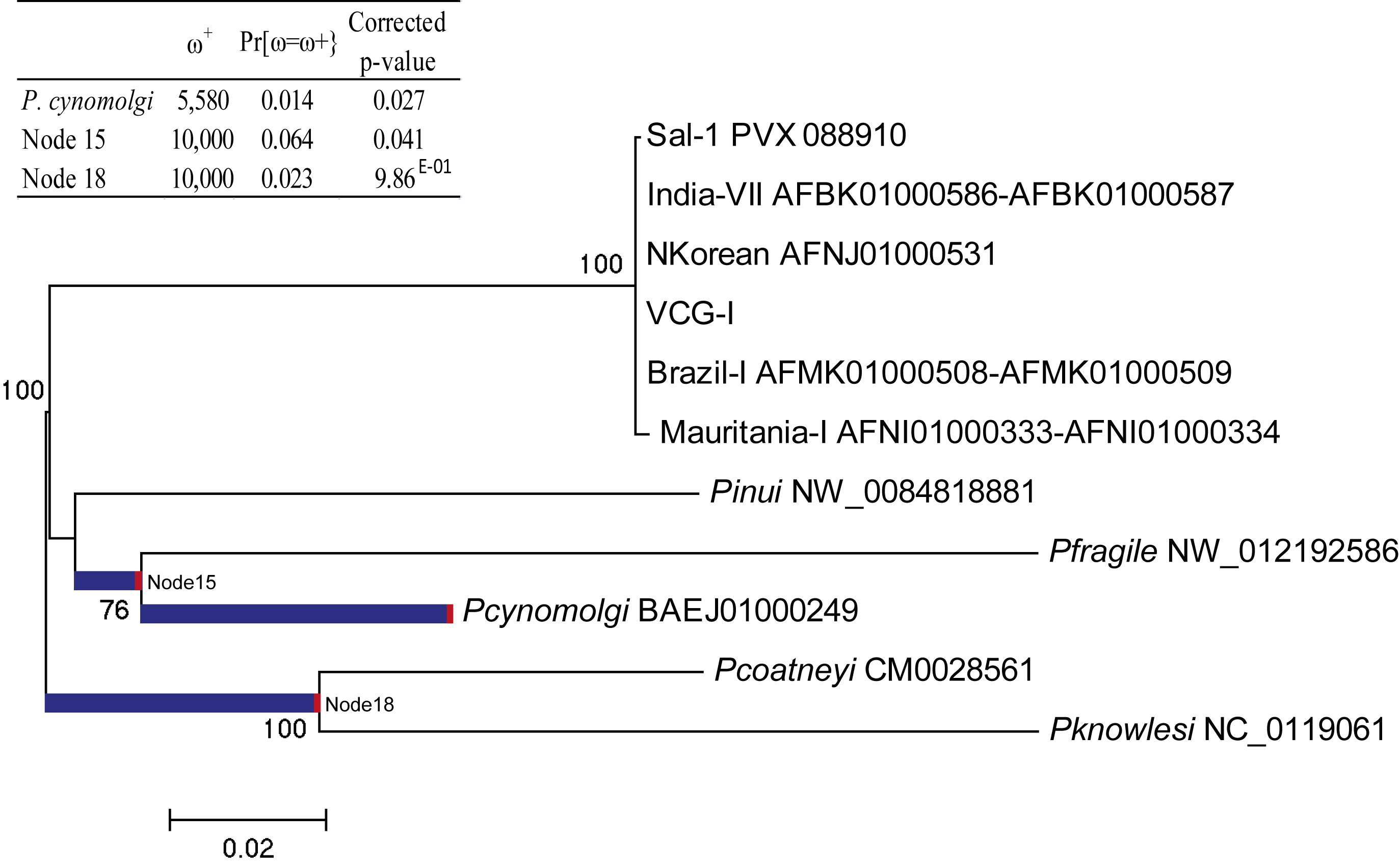

Supplement: Supplementary file 2 — Lineage-specific positive selection. Branches under positive episodic selection were identified by using the REL-site branch method. Episodic selection acts very quickly and involves a switch from negative to positive natural selection and back to negative and might enable adaptation to a new host. Phylogeny was inferred in MEGA v6 by the maximum likelihood method using the GTR + G evolutionary model. ω+ model: ω rate values. Pr [ω = ω +]: percentage of sites evolving under positive selection. P-value corrected for multiple tests using the Holm-Bonferroni method. (TIF 470 kb) [file 13071_2017_2183_MOESM2_ESM.tif]

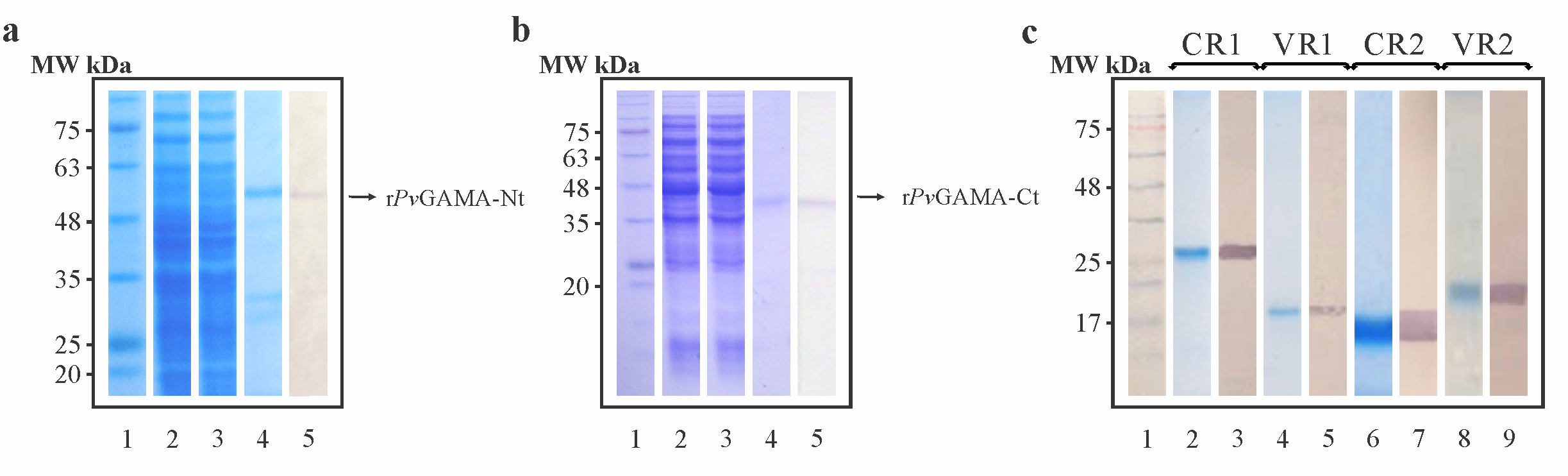

Supplement: Supplementary file 3 — Obtaining recombinant proteins. a, b Recombinant GAMA protein expression and purification. Lanes 2–3 show non-induced and induced cell lysate, respectively. Lanes 4–5 show purified rPvGAMA-Nt and -Ct stained with Coomassie blue or analysed by western blot using anti-polyhistidine antibodies, respectively. c Purifying conserved (CR1 and CR2) and variable (VR1 and VR2) PvGAMA regions. Lanes 2, 4, 6 and 8 show purified recombinant proteins and lanes 3, 5, 7 and 9 show western blot detection. The proteins’ molecular markers are indicated in Lane 1 on all figures. (TIF 5327 kb) [file 13071_2017_2183_MOESM3_ESM.tif]

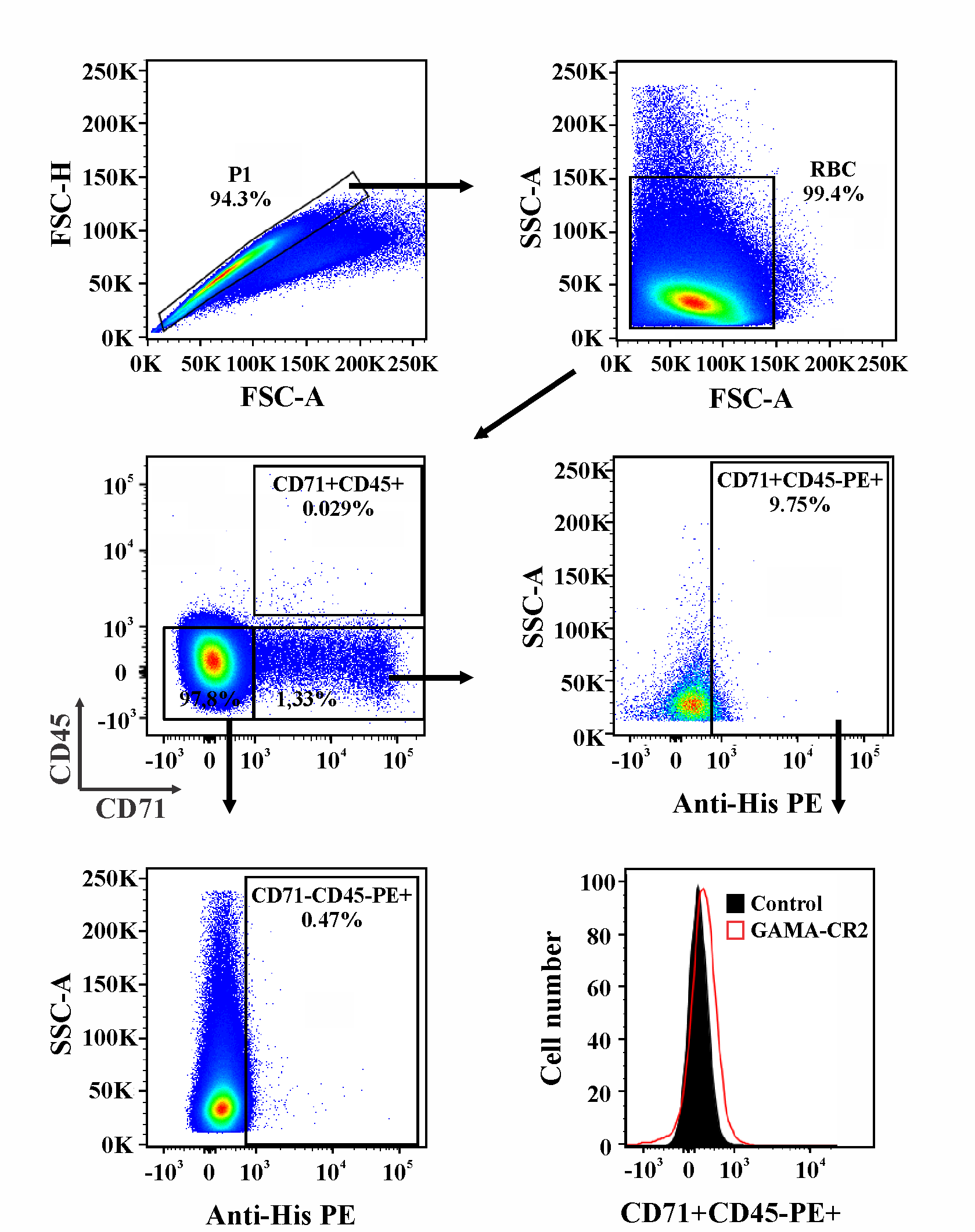

Supplement: Supplementary file 4 — Selection strategy for immature and mature erythrocyte populations. The doublets were excluded by plotting FSC-H against FSC-A. Cells were then selected by their granularity, using an SSC-A vs FSC-A cytogram. The CD45 vs CD71 signal was plotted for selecting reticulocyte (CD71 + CD45-) and mature erythrocyte (CD71-CD45-) populations and omitting activated lymphocytes (CD71 + CD45+). The percentage of cells having bound protein was calculated using the PE signal (CD71 + CD45-PE+). A representative histogram from three independent experiments analysing the PE signal for the CR2 binding assay compared to control is also shown. (TIF 10448 kb) [file 13071_2017_2183_MOESM4_ESM.tif]
